# Identification of coexistence of *BRAF V600E* mutation and *EZH2* gain specifically in melanoma as a promising target for combination therapy

**DOI:** 10.1186/s12967-017-1344-z

**Published:** 2017-12-04

**Authors:** Huan Yu, Meng Ma, Junya Yan, Longwen Xu, Jiayi Yu, Jie Dai, Tianxiao Xu, Huan Tang, Xiaowen Wu, Siming Li, Bin Lian, Lili Mao, Zhihong Chi, Chuanliang Cui, Jun Guo, Yan Kong

**Affiliations:** 1 The Key Laboratory of Carcinogenesis and Translational Research (Ministry of Education/Beijing), Department of Renal Cancer and Melanoma, Peking University Cancer Hospital & Institute, Beijing, China; 2 Department of Renal Cancer and Melanoma, Peking University Cancer Hospital and Institute, 52 Fucheng Road, Haidian District, Beijing, 100142 China

**Keywords:** *EZH2* gain, *BRAF V600E* mutation, Combination therapy, Melanoma

## Abstract

**Background:**

Coexistence of enhancer of zeste homolog 2 (*EZH2*) and *BRAF* gene aberrations has been described in many cancer types. In this study, we aim to explore the coexistence status of *BRAF V600E* mutation and the copy number variation of *EZH2* and explore the potential of this combination as a therapeutic target.

**Methods:**

A total of 138 cases of melanoma samples harboring *BRAF V600E* mutation were included, and *EZH2* copy numbers were examined by QuantiGenePlex DNA Assays. Clinical pathological distinction between patient groups with or without *EZH2* amplification (hereafter referred to as *EZH2* gain) was statistically analyzed. The sensitivity of melanoma cell lines and patient-derived xenograft (PDX) models containing *BRAF V600E* mutation with or without *EZH2* gain to vemurafenib (*BRAF* inhibitor), GSK2816126 (*EZH2* inhibitor) and a combination of both agents was evaluated.

**Results:**

In our cohort, the coexistence rate of *BRAF V600E* mutation and *EZH2* gain was up to 29.0%, and significant differences in overall survival and disease-free survival were found between no *EZH2* copy number gain and gain groups (*P* = 0.038, *P* = 0.030), gain and high *EZH2* copy number gain groups (*P* = 0.006, *P* = 0.010). Combination with *BRAF* and *EZH2* inhibition showed better inhibitory efficacy in melanoma prevention compared with vemurafenib monotherapy. More importantly, this improved therapeutic effect was observed especially in melanoma cell lines and PDX models containing concurrently *BRAF V600E* mutation and *EZH2* gain.

**Conclusions:**

Coexistence of *BRAF V600E* mutation and *EZH2* gain is rather prevalent in melanoma. Our findings provided evidence for the feasibility of combination therapy with *EZH2* and *BRAF* inhibitors in melanoma with concurrent *BRAF V600E* mutation and *EZH2* gain.

**Electronic supplementary material:**

The online version of this article (10.1186/s12967-017-1344-z) contains supplementary material, which is available to authorized users.

## Background

The incidence of melanoma, one of the most malignant cancer, is increasing worldwide [1]. The aggressiveness of melanoma is dependent on the high metastatic potential of melanoma cells, which can still not be effectively targeted despite recent progresses in targeted therapy and immunotherapy [2].

The mutation rates of *BRAF V600E* in Caucasians and Asians are approximately 50 and 25%, respectively [3]. Vemurafenib, a *BRAF* inhibitor, has been shown to improve outcomes in the majority of melanoma patients harbouring *BRAF V600E* mutation, with a median overall survival (OS) of approximately 16 months [4]. However, most patients treated with vemurafenib show disease progression within 6–8 months due to invariable drug resistance [4–12]. Recently, combined therapy has significantly improved response rates, along with progression-free and overall survival compared with single agent, such as vemurafenib plus trametinib, dabrafenib (*BRAF* inhibitor) plus trametinib, vemurafenib plus pembrolizumab and nivolumab plus ipilimumab [13–15]. However, despite rapid early response and high response rate to these combination therapeutic regimens, progression of disease occurs at a median of 11 months, with few patients remaining progression-free beyond 15 months [16], thus novel combination targets are urgently needed to be found.

The enhancer of zeste homolog 2 (*EZH2*) gene, located on chromosome 7q36.1, is adjacent to the *BRAF* gene which located on chromosome 7q34. Abnormalities in these two genes often coexist in various types of cancer, including papillary thyroid carcinoma [17]. *EZH2* is core component of the polycomb repressive complex 2, which catalyzes trimethylation of lysine 27 in histone 3 (H3K27me3), inducing chromatin compaction and preventing the transcription of target genes which are mostly tumor suppressor genes [18]. Dysregulation of the *EZH2* gene has been observed in several types of cancers, including lung, breast, and prostate cancer [17, 19, 20]. Growing evidence demonstrates that *EZH2* is imperative for cancer initiation, development, progression, metastasis, and drug resistance. Therefore, *EZH2* is currently considered a promising drug target, and multiple inhibitors of *EZH2* have been developed, some of which are in clinical trials [21]. Moreover, recent studies have shown that *EZH2* also plays a critical role in the proliferation and survival of melanoma [19, 22–28].

*EZH2* gain-of-function mutations often occur concurrently with the *BRAF V600E* mutation in melanoma [29]. Knockdown of *BRAF* directly downregulates *EZH2* gene expression in melanoma cells [30] and prostate cancer [31]. Therefore, there may be a close relationship between aberrant *EZH2* gene expression and the *BRAF V600E* mutation, thus highlighting *EZH2* as a promising combination therapeutic target with vemurafenib for targeted therapy.

Herein, to reveal the potential of this combination as a therapeutic target, we will explore the coexistence status of *BRAF V600E* mutation and *EZH2* gain in large-scale melanoma samples and show whether or not a combination of *BRAF* and *EZH2* inhibition is effective for inhibition of melanoma cell growth in vitro and in vivo.

## Methods

### Patients and tumor tissue samples

This study involved samples from primary lesions of 138 patients with melanoma, who were hospitalized from January 2007 to January 2015 at Beijing Cancer Hospital & Institute. The diagnosis of melanoma was confirmed by hematoxylin and eosin staining and immunohistochemistry. BRAF V600E mutation status was verified by Sanger sequencing. This study was approved by the Medical Ethics Committee of the Beijing Cancer Hospital & Institute and was conducted according to the principles of the Declaration of Helsinki.

### DNA preparation and *BRAF V600E* mutation screening

Genomic DNA was extracted from formalin-fixed, paraffin-embedded sections using a QIAamp DNA FFPE Tissue Kit (Qiagen, Valencia, CA, USA). To detect the BRAF V600E mutation, we amplified exon 15 of the *BRAF* gene by polymerase chain reaction (PCR) in at least two separate preparations of genomic DNA. The primer sequences were as follows: 15F1, 5′-AAACTCTTCATAATGCTTGCTC-3′; 15R1, 5′-TAATCAGTGGAAAAATAGCCTC-3′; 15F2, 5′-CTTGCTCTGATAGGAAAATG-3′; and 15R2, 5′-AGCCTCAATTCTTACCATCC-3′. All *BRAF* sequencing reactions were performed from forward and reverse directions and in duplicate. We purified PCR products with QIAquick (Qiagen) and directly sequenced them using Big Dye Terminator sequencing chemistry on an ABI 3130 automated sequencer (Applied Biosystems, Foster City, CA, USA). All mutations were confirmed by repeat bidirectional sequencing on the ABI sequencer.

### QuantiGene Plex DNA assay

Tissue homogenates were prepared using the QuantiGene sample processing kit for formalin-fixed paraffin-embedded tissues (FFPE; Panomics of Affymetrics, Santa Clara, CA, USA) according to the manufacturer’s instructions. Briefly, 5–8 pieces of deparaffinized sections (4–10 μm) were incubated with 150 μl homogenizing solution supplemented with 1.5 μl of proteinase K (50 μg/μl) at 65 °C for 6 h. The tissue homogenate was separated from debris by brief centrifugation and transferred to a new tube.

The branched DNA (bDNA) assay was performed using the QuantiGene Plex DNA kit (Panomics) according to the manufacturer’s instructions. Briefly, the homogenate DNA was sheared using Covaris S2 (Covaris, Woburn, MA, USA) with the following settings: duty cycle 5%, intensity 3, cycles/burst 200, 80 s. For each assay well, 40 μl homogenate was denatured with 2.5 M NaOH (final concentration 0.18 M) in the presence of a DNA probe. The neutralized tissue homogenate was transferred to each well of hybridization plates containing the working bead mix. All samples were in processed in duplicates. The hybridization plate was sealed and incubated at 54 ± 1 °C in shaking incubator (600 rpm) for 18–22 h. The unbound samples were washed away using the Bio-plex Pro II wash station (Bio-Rad, Hercules, CA, USA). Then, the beads were sequentially hybridized with DNA pre-amplifier, DNA amplifier, labeled probe, and SAPE (streptavidin-conjugated R-phycoerythrin). Fluorescence intensities were measured using the Bio-plex 100 system (Bio-Rad).

The mean fluorescence intensities of the duplicates were calculated for all genes. Background values were subtracted from each probe set signal. Values of tested genes were normalized to the geometric means of Rpph1, Rpp30, and Rplp0. For each test gene, the normalized signal was divided by the signal of the reference DNA sample (G1521, Promega, Madison, WI, USA) and the values were multiplied by the known copy number (usually 2 copies) of each gene in the reference genome.

### EZH2 gene copy number variation analysis by real-time PCR

To validate the results of QuantiGenePlex DNA Assays, the copy numbers of *EZH2* were further quantified by TaqMan Copy Number Assays (Applied Biosystems, ThermoFisher, Waltham, MA, USA). A TaqMan probe targeting the *Rnasep* gene was used as a control. The *EZH2* Tagman probe (4400291) was purchased from Invitrogen (ThermoFisher). Quantitative real-time PCR was performed using an ABI 7500 FAST real-time PCR system (Applied Biosystems). Copy numbers were then determined using CopyCaller v2.0 software (Applied Biosystems) with the comparative Ct (ΔΔCt) method. The *EZH2* copy numbers for melanoma samples were calculated by dividing the sample values by the control values (benign nevus). No gain was considered for copy number ≤ 2, whereas gain was considered for copy number ≥ 2 and high gain was considered for copy number ≥ 4.

### Cell culture

SK-MEL-5 (cat. no. HTB-70), WM-1664 (cat. no. CRL-1676), WM-115 (cat. no. CRL-1675), A2058 (cat. no. CRL-11147), and A375 cells were obtained from American Type Culture Collection (ATCC) and cultured at 37 °C in Dulbecco’s modified Eagle’s medium (DMEM; Invitrogen, ThermoFisher) supplemented with penicillin, streptomycin (Invitrogen), and 10% fetal bovine serum (HyClone, GE Healthcare, Logan, UT, USA).

### Western blots

All cells and tumor tissues of PDX models were lysed using the PhosphoSafe extraction reagent (Millipore). Supernatants were collected by centrifugation. Western blotting analysis of protein complexes were performed with the following antibodies: *EZH2* (1:1000; Cell Signaling Technology, Danvers, MA, USA), H3K27me3 (1:1000; Cell Signaling Technology, Danvers, MA, USA), GAPDH (1:10,000; abcam, Cambridge, UK).

### Immunohistochemistry (IHC)

IHC analysis of EZH2, P-ERK and P-AKT protein was performed using anti-EZH2 polyclonal rabbit antibodies, anti-P-AKT polyclonal rabbit antibodies and anti-P-ERK polyclonal rabbit antibodies (Cell Signaling Technology, Danvers, MA, USA) at a dilution of 1:1000, followed by a standard avidin–biotin detection protocol using diaminobenzidine. Hematoxylin-counterstained slides were mounted and examined to determine the intensity of staining. The staining intensity was scored as −, +, ++, or +++ (with − indicating negative staining, and +++ indicating the strongest staining) independently by three pathologists.

### Cell cycle and apoptosis assays

Cell cycle and apoptosis assays were performed using A2058 cells at 24 or 48 h after exposure to vemurafenib, GSK126, or both vemurafenib and GSK126 using a BD Pharmingen (BD Biosciences, Sparks, MD, USA) and Annexin V FITC Apoptosis Detection Kit (DOJINDO, Shanghai, China) according to the manufacturers’ protocols. Analysis was performed on a FACS Calibur flow cytometer (BD Biosciences).

### Effects of *EZH2* and *BRAF* inhibitors on proliferation

GSK126 and vemurafenib were purchased from Selleck Chemicals (Houston, TX, USA). All inhibitors were dissolved at 10 mM in dimethylsulfoxide (DMSO) as stock solutions. After treatment with various concentrations of inhibitors or DMSO for 24 h, cell proliferation was evaluated by CellTiter-Glo Luminescent Cell Viability Assays (Promega, Madison, WI, USA) according to the manufacturer’s instructions.

### Calculation of the combination index (CI)

The combined activity of vemurafenib and GSK126 was determined by calculating the CI for both compounds in A2058 and WM115 cells with the following equation in Compusyn software: CI = (D1/Dx1) + (D2/Dx2) + α [(D1 × D2)/(Dx1 × Dx2)], where Dx1 is the concentration of drug 1 required to produce 50% cell death alone, D1 is the concentration of drug 1 required to produce 50% cell death in combination with D2, Dx2 is the concentration of drug 2 required to produce 50% cell death alone, D2 is the concentration of drug 2 required to produce 50% cell death in combination with D1, and α is 0 for mutually exclusive or 1 for mutually nonexclusive modes of drug action. The results are interpreted as: 0 < CI < 1 indicates synergism; CI = 1 indicates an addictive effect; and CI > 1 indicates antagonism.

### Patient-derived xenograft (PDX) model and treatment

Fragments of patient-derived melanoma tissues bearing *BRAF V600E* mutation were cut into fragments and then subcutaneously inoculated into a 5 week-old NOD/SCID (non-obese diabetic and severe combined immunodeficiency) female mouse (4–6 week-old, 18–22 g-weight) to establish the PDX model. When the tumor size reached approximately 1 cm^3^, the mice were sacrificed, and tumor tissues were separated and re-inoculated into new mice. 2 PDX models (detailed information are listed in Additional file 1: Table S1) were finally established: PDX 001 (concurrently containing *BRAF V600E* mutation and *EZH2* gain), and PDX 002 (containing *BRAF V600E* mutation alone).

When the tumor size reached approximately 600 mm^3^, each type of PDX models were randomized (treatment arm versus control arm; n = 5) into four groups: group 1 received oral vemurafenib (30 mg/kg) twice a day; group 2 received GSK126 (50 mg/kg) intraperitoneally once a day; group 3 received a combination of the two agents with doses and administration methods similar to those introduced in single treatment; and group 4 served as a negative control and received vehicle once a day by intraperitoneal injection of 100 µl corn oil and oral gavage of 100 µl PBS. Body weights and the lengths and widths of tumors were measured every 3 days using scales and calipers, and the tumor volume was calculated as ([length × width^2^] × 0.5). After 14 days, the mice were sacrificed, and the tumors were fixed in 10% formalin for histological and immunohistological analysis. All animal care and experimental procedures were carried out in accordance with the Animal Care Ethics guidelines approved by the Medical Ethics Committee of Beijing Cancer Hospital & Institute.

### Statistical analyses

Statistical analyses were performed using SPSS 22.0 software. Continuous data, such as age and lesion thickness, were described using means ± standard deviations (SDs) for normally distributed data. The correlations between aberration status and clinical parameters were evaluated by Chi square test or Fisher’s exact test. Kaplan–Meier estimates of time-to-event OS were calculated. Log-rank tests were used to estimate the statistical significance between the time-dependent outcomes of OS. Cox hazard proportion models were used to estimate the hazard ratios (HRs) and corresponding 95% interval confidences (CIs). All statistical analyses were two-sided, and differences with *P* values of less than 0.05 were considered statistically significant.

## Results

### *EZH2* gain in *BRAF V600E*-mutated melanoma

Among the 138 patients with *BRAF V600E*-mutated melanoma, 40 cases (29.0%) showed *EZH2* gain (Table 1). Additionally, we detected the *EZH2* gain frequency in melanoma subtypes and found that the frequencies of *EZH2* gain in acral melanoma, mucosal melanoma, melanoma on skin with chronic sun-induced damage (CSD) and melanoma on skin without chronic sun-induced damage (non-CSD) were 21.9, 38.9, 25.0 and 33.3%, respectively (Table 1).

**Table 1:** *EZH2* gain in *BRAF V600E* mutated melanoma

Melanoma subtypes	Number of cases	Number of cases with EZH2 gain (%)
Acral melanoma	41	9 (22.5)
Mucosal melanoma	18	7 (17.5)
CSD	36	9 (22.5)
Non-CSD	39	13 (32.5)
Total	138	40 (29.0)
P value		0.346

Altogether, our data suggested that the coexistence of *EZH2* gain and the *BRAF V600E* mutation was prevalent in melanoma, especially mucosal subtype.

### Correlation of *EZH2* gain with clinicopathological features in patients with *BRAF V600E*-mutated melanoma

A summary of the correlations between *EZH2* gain and clinical characteristics are shown in Table 2. The mean age (*P* = 0.312) and gender (*P* = 0.553) were not significantly different between melanoma patients harbouring *BRAF V600E* mutation with or without *EZH2* gain.

**Table 2:** Correlation of *EZH2* gain to clinicopathologic features of *BRAF V600E* mutated melanomas

Clinicopathologic feature	*EZH2* gain		
	Positive	Negative	*P* value^a^
Age (year)	51.5 ± 12.6	51.4 ± 13.0	0.312
Gender N (%)			0.553
Man	19 (47.5)	52 (53.1)	
Female	21 (52.5)	46 (46.9)	
Thickness (mm)			0.907
< 1	0 (0.0)	0 (0.0)	
1–2	3 (27.3)	12 (27.3)	
2–4	6 (38.6)	17 (38.6)	
> 4	5 (34.1)	15 (34.1)	
Ulceration N (%)			0.001
Yes	12 (35.3)	60 (69.0)	
No	22 (64.7)	27 (31.0)	
Primary site N (%)			0.503
Acral	9 (22.5)	32 (32.7)	
Mucosal	7 (17.5)	11 (11.2)	
CSD	9 (22.5)	27 (27.6)	
Non-CSD	13 (32.5)	26 (26.5)	
Unknown primary	2 (5.0)	2 (2.0)	
TNM stage N (%)			0.071
I	4 (10.0)	3 (3.1)	
II	5 (12.5)	29 (29.6)	
III	17 (42.5)	31 (31.6)	
IV	14 (35.0)	35 (35.7)	

^a^For evaluation of age, the unpaired t or t’ tests were used. For evaluation of gender, ulceration and stages, the Chi square tests or Fisher’s exact tests were used. For evaluation of thickness, Mann–Whitney U tests were used

**Fig. 1:**
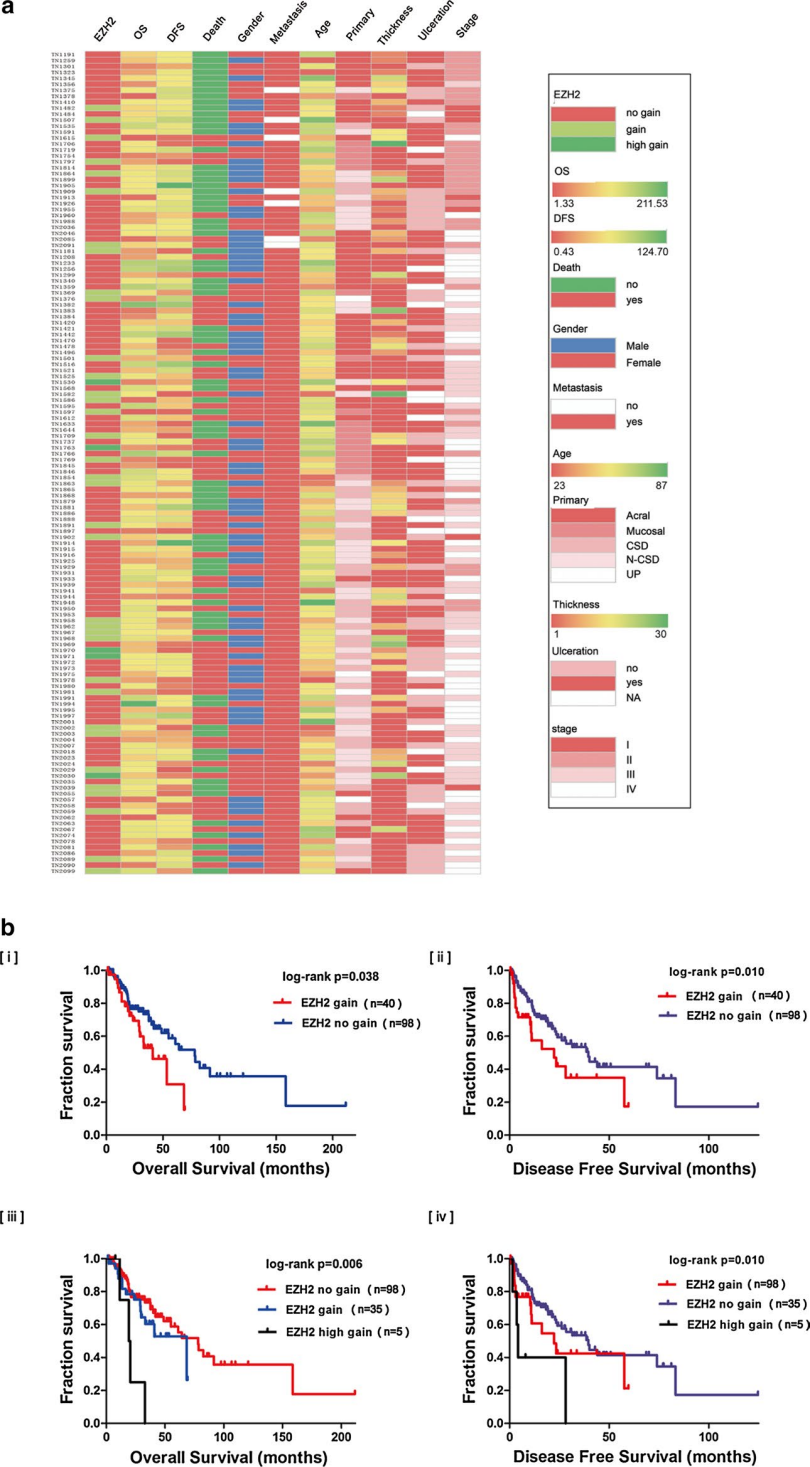
*EZH2* gain was an adverse prognostic factor of *BRAF V600E* mutated melanoma. **a** Correlation of *EZH2* gain with clinicopathological features in patients with *BRAF V600E*-mutated melanoma was shown by heat map. **b** Comparison of the OS and DFS of patients with different *EZH2* gain levels was conducted by the Kaplan–Meier method. [i, ii] No *EZH2* copy number gain and gain groups. [iii, iv] No EZH2 copy number gain, gain and high gain groups

Thickness, ulceration, and stage are important clinical characteristics that can be used to predict the prognosis in patients with melanoma. In our cohort, the data for stage (138 cases), thickness (59 cases), and ulceration (122 cases) were available for further analysis (Fig. 1a).

The average thickness of the melanoma lesions in 59 patients with the *BRAF V600E* mutation was 4.8 mm, consistent with the observation that thickness in Chinese patients is thicker than that in Caucasians [1]. Additionally, the average thickness of lesions in melanomas harboring *EZH2* gain was 5.525 ± 3.475 mm, whereas that in lesions without *EZH2* gain was 4.65 ± 3.35 mm; there was no significant difference between these two groups (*P* = 0.907).

Ulceration is another adverse prognostic factor in melanoma. In our cohort, the overall ulceration rate for the 122 available cases was 59.5% (72/121), and the ulceration rates in acral, mucosal, CSD, and non-CSD melanomas were 61.0% (25/41), 44.4% (8/18), 30.6% (11/36), 41.0% (16/39), respectively. The ulceration rate in melanomas harboring *EZH2* gain was 35.3%, whereas in melanomas without *EZH2* gain was 69% (*P* = 0.001). Thus, *EZH2* gain did not appear to be correlated with the formation of ulceration, further studies with larger sample sizes are needed to confirm this result.

Among melanoma patients containing concurrently *BRAF V600E* mutation and *EZH2* gain, the percentages of patients with stages I, II, III and IV were 10% (four cases), 12.5% (five cases), 42.5% (17 cases), and 35.0% (14 cases), respectively (*P* = 0.071); these data were not significantly different from those in patients without *EZH2* gain. However, the percentage of patients with stages III and IV in the *EZH2* gain group was 77.5%, which was substantially higher than that in the *EZH2* no gain group (67.3%); thus, further studies with larger sample sizes are needed to confirm the differences in disease stage between the two groups.

Moreover, correlation of EZH2 gain with clinicopathological features among three groups (no gain, gain, high gain) were also performed, tables were provided in Additional file 2.

### Prognostic significance of EZH2 gain for OS and DFS in patients with BRAF V600E mutated melanoma patients

The thickness and ulceration of primary melanoma lesions and the stage of the disease are adverse prognostic factors. Consistent with previous reports, we found that the overall survival (OS) of patients with thicker lesions and more advanced-stage disease was significantly shorter than those with thinner lesions (*P* = 0.023) and early-stage disease (*P* < 0.001), respectively. In addition, in our cohort, the OS of patients with mucosal melanoma and unknown primary melanoma was significantly shorter than that of patients with other subtypes (*P* < 0.001; data not shown).

Next, we analyzed the prognostic significance of *EZH2* gain for overall survival (OS) and disease-free survival (DFS) in melanoma patients harbouring *BRAF V600E* mutation. Significant differences in overall survival and disease-free survival were found between no *EZH2* copy number gain and gain groups (*P* = 0.038, HR 0.50, 95% CI 0.02–0.61; *P* = 0.030, HR 0.49, 95% CI 0.26–0.94), gain and high *EZH2* copy number gain groups (*P* = 0.006, *P* = 0.010) (OS median value: no gain group: 78.1 months; gain group: 40.8 months; high gain group: 31.3 months; DFS median value: no gain group: 39.5 months; gain group: 22.3 months; high gain group: 11.1 months) (Fig. 1b).

In univariate Cox analysis, the clinicopathologic factors, such as TNM stage and EZH2 amplification, may be of prognostic significance for melanoma patients; For multivariate Cox regression assay, TNM stage is independent prognostic factors for OS (Additional file 3).

Taken together, our findings indicated that *EZH2* gain may be an adverse prognostic biomarker in BRAF V600E-mutated melanoma.

### *EZH2* copy number levels and corresponding *EZH2* protein levels in four *BRAF V600E*-mutated cell lines, PDX models and patient tissues

**Fig. 2:**
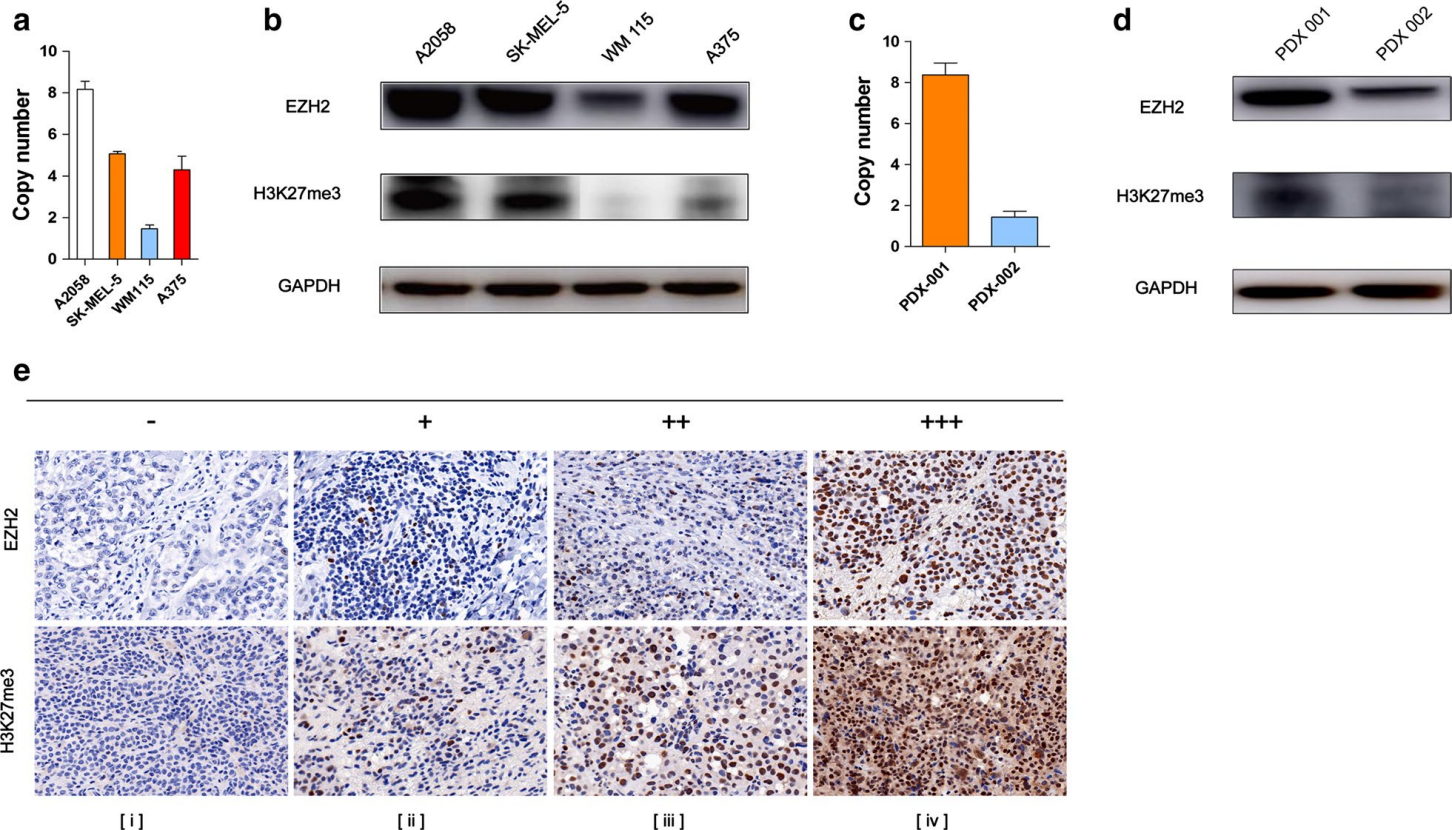
*EZH2* copy number levels and corresponding protein levels in cell lines, PDX models and patient tissues. **a**, **c** qRT-PCR analysis was used to detect *EZH2* copy number levels in four *BRAF*-mutated cell lines and PDX models. **b**, **d** Immunoblots was conducted to determine the protein levels of *EZH2* in four *BRAF*-mutated cell lines and PDX models. GAPDH serves as loading control. **e** Representative images from *EZH2* staining of the same samples described in Fig. 1a magnification, ×200. Scale bars, 100 um

*EZH2* copy number levels, and corresponding western blotting results for four cell lines and two PDX models are shown in Fig. 2a–d. The copy number decreased in the order of A2058 > SK-MEL-5 > A375 > WM115 in cell lines and PDX-001 > PDX-002 in mouse models, corresponding protein levels were roughly consistent with copy number levels.

We also examined EZH2 protein expression and H3K27me3 levels by IHC (Fig. 2e) which supported our previous detected *EZH2* copy number aberrations. H3K27me3 levels reflected the active status of *EZH2* protein.

These findings indicated that EZH2 protein expression was positively correlated with copy number alterations.

### Inhibitory effect of combination on *BRAF V600E*-mutated cell lines

**Fig. 3:**
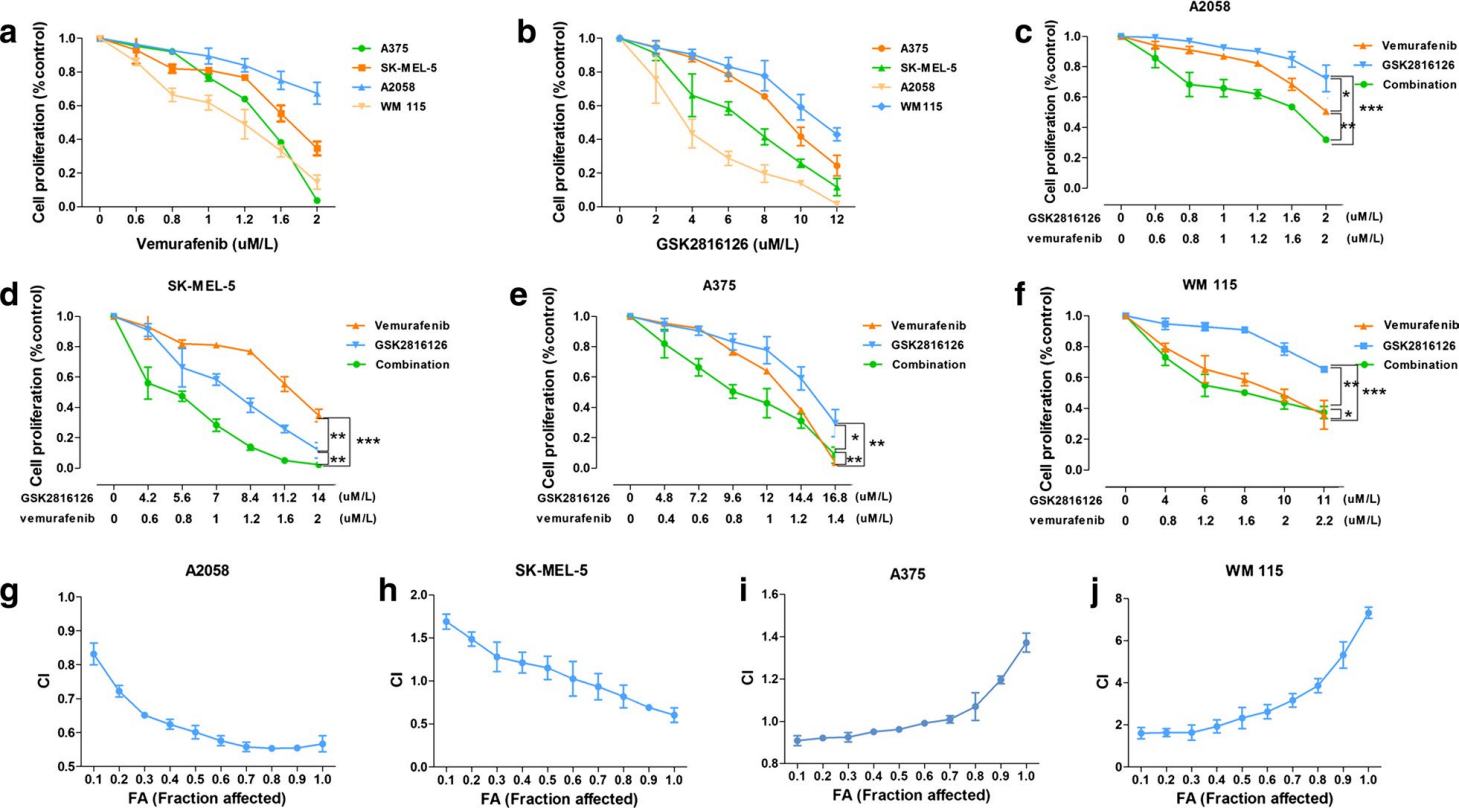
Therapeutic effects of combination of *EZH2* and *BRAF* inhibition in vitro. Four *BRAF* mutated cell lines (A375, SK-MEL-5, A2058, WM 115) was treated with indicated concentrations of inhibitors for 48 h. The proliferation of cells was evaluated by Cell Tilter method. **a**, **b** Inhibitory effect of monotherapy with vemurafenib and GSK126 respectively. **c**–**f** Comparison of inhibitory effect of the combined treatment with vemurafenib and GSK126 and single agent alone in A2058, SK-MEL-5, A375 and WM 115 cell growth. **g**–**j** The calculated CI (Combination Index) of combination therapy in A2058, SK-MEL-5, A375 and WM 115 cell lines. The results were presented as mean ± SD of three independent experiments. The statistical significance of the growth curves was evaluated by Mauchly’s test of sphericity (*P < 0.05; **P < 0.01; ***P < 0.001)

A2058, WM115, A375, and SK-MEL-5, all four BRAF V600E-mutated cell lines were evaluated to determine the efficacy of GSK126 and vemurafenib by analysis of cell proliferation rates in vitro. The concentration–response curve for each cell line is shown in Fig. 3a, b. For vemurafenib, WM115 cells (half-maximal inhibitory concentration [IC_50_] = 1.7 μM) were the most sensitive, and A2058 cells (IC_50_ = 4 μM) were the least sensitive. For GSK126, A2058 cells (IC_50_ = 4 μM) were the most sensitive, whereas WM115 cells (IC_50_ = 10 μM) were the least sensitive. The IC_50_ values were then used to generate fixed ratios for subsequent combination treatment regimens and calculation of CIs.

Combination therapy with these two agents was also performed in all four cell lines. The concentration–response curve is shown in Fig. 3c–f. For evaluation of the combination of vemurafenib and GSK126, the cells were exposed to medium containing both drugs concurrently at a fixed ratio approximating their individual IC_50_s for 48 h to ensure that both agents contributed equally to the observed antiproliferative effects. As shown in Fig. 3c, for A2058 cells, the drug combination showed increased inhibitory efficacy compared either agent used alone; for SK-MEL-5 cells, the drug combination showed advantages only under high doses condition (vemurafenib: 0.8 μM/l, GSK126: 5.6 μM/l); for A375 cells, combination therapy showed advantages only under low doses condition (vemurafenib: 0.8 μM/l, GSK126: 9.6 μM/l). However, in WM115 cells, combination therapy showed no obvious advantage. To further elucidate the interaction patterns of these two drugs, CIs and fractional cell growth inhibition (Fa) values were calculated (Fig. 3g–j). CIs of significantly less than 1 were obtained in A2058 cells (mean CI at Fa 0.5 = 0.885), SK-MEL-5 cells (mean CI at Fa 0.5 = 1.260) under high doses condition and A375 cells (mean CI at Fa 0.5 = 0.972) under low doses condition, demonstrating that the two drugs interacted synergistically to inhibit cell growth totally or partially. In contrast, values more than 1 were obtained in WM115 cells, indicating that the two drugs interacted antagonistically (mean CI at Fa 0.5 = 1.370) (Compusyn reports can be seen in Additional files 4, 5, 6, 7 and 8). After treatment, P-AKT protein levels were detected in GSK126 monotherapy groups of 4 cell lines, we found that *EZH2* inhibition was associated with downregulation of P-AKT levels (Additional file 9: Figure S3).

This result indicated that combination therapy could achieve better anticancer efficacy only in melanoma cell lines with concurrent *BRAF V600E* mutation and *EZH2* gain, and the effects of GSK126 treatment may involve inhibition of PI3K signaling pathway.

### Variations in cell cycle distributions and apoptosis rates in cells after combination therapy

We next examined whether the suppression of cell proliferation by combination therapy was caused by cell cycle arrest or apoptosis. A2058 cells were used for further study.

**Fig. 4:**
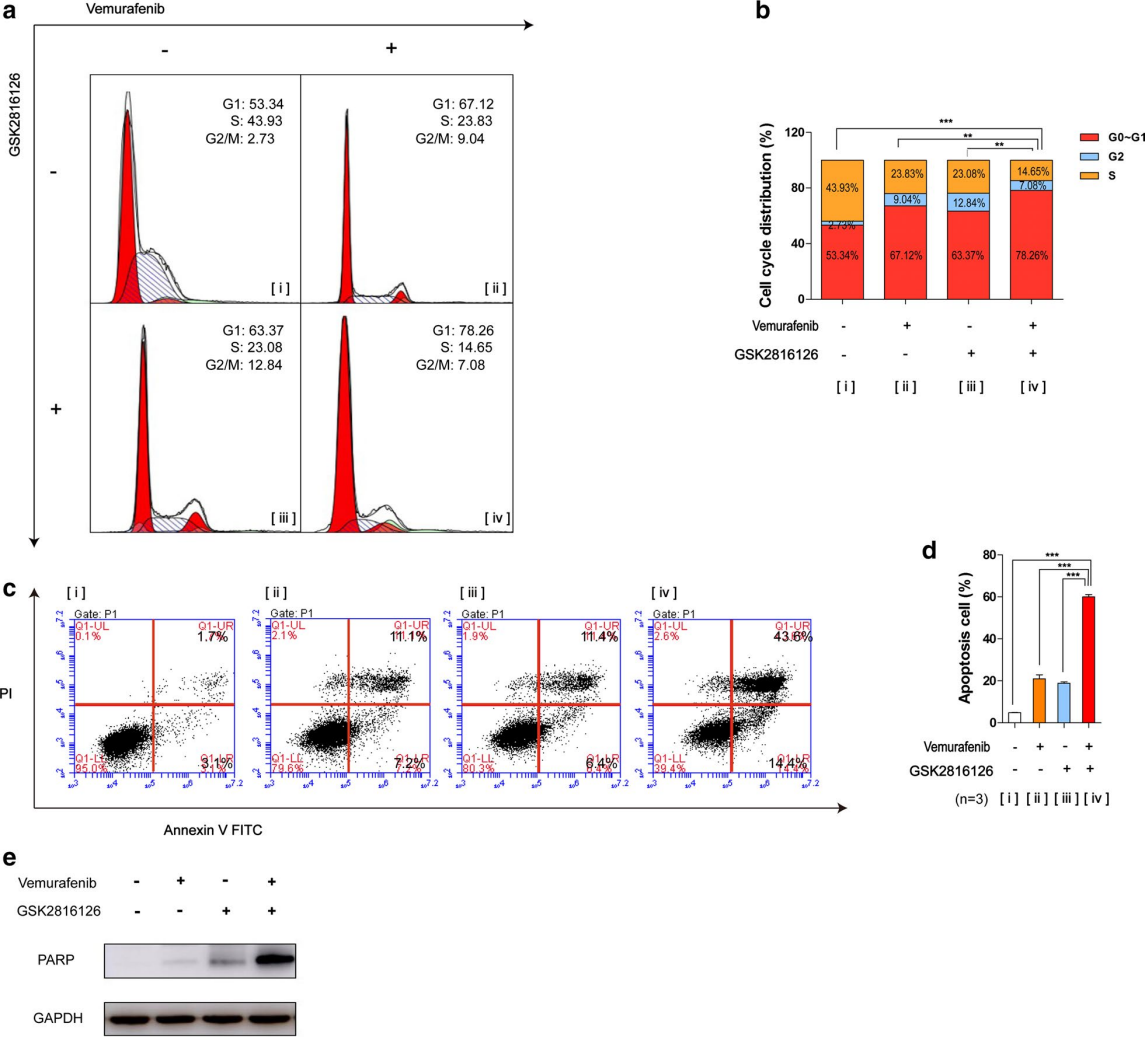
Variations in cell cycle distributions and apoptosis rates in cells after combination therapy. Combined treatment induced obvious G1 phase cell cycle arrest and apoptosis. **a** A2058 cells were treated with indicated concentrations of combination therapy and monotherapy for 48 h and subsequent to PI staining and flow cytometry analysis. **b** Graphs of the analysis were shown. The results were presented as mean ± SD (n = 3) (*P < 0.05; **P < 0.01; ***P < 0.001). **c** A2058 cells were treated with indicating concentrations of combination therapy and monotherapy for 24 h. Apoptotic cells were detected with Annexin V-FITC and PI double staining followed by flow cytometry analysis. **d** Graphs of the analysis were shown. Mean ± SD (n = 3). [i] Control, [ii] monotherapy with vemurafenib, [iii] monotherapy with GSK126, [iv] combination therapy. **e** Immunoblots was conducted to determine the protein levels of PARP. GAPDH serves as loading control

The cell cycle phase distribution of cells treated with vemurafenib (1 μM), GSK126 (1 μM), and combined treatment (1 μM each) for 48 h was detected by flow cytometry. We found that compared with the monotherapy group, cells treated with combined treatment showed a significant increase in the proportion of cells at G_1_ phase and a reduction in the proportion of cells in the S phase (*P* < 0.05; Fig. 4a, b). The percentages of cells in the G_1_ phase following combination therapy increased by approximately 24.9, 11.14, and 14.89% compared with the control, vemurafenib, and GSK126 groups, respectively. In addition, Sub-G0 population detected by flow cytometry showed that combination therapy caused more obvious cell death than monotherapy groups (Additional file 9: Figure S1).

The apoptosis rates in A2058 cells treated with vemurafenib (1.2 μM), GSK126 (1.2 μM), or both drugs (1.2 μM each) for 24 h were detected by flow cytometry with Annexin V-FITC/propidium iodide (PI) double staining. We found that combined treatment caused obvious apoptosis effects, with apoptosis rates of 53.2, 39.7, and 40.3% more than those of the control, vemurafenib, and GSK126 groups, respectively. Results of western blotting for PARP (apoptosis biomarker) further confirmed this conclusion (Fig. 4e). Then, we probed this result in other three cell lines, we found that combination therapy caused more apparently cell apoptosis especially in cells harboring *EZH2* gain. (A2058, SK-MEL-5 and A375) (Additional file 9).

Taken together, these results indicated that vemurafenib and GSK126 could function synergistically to induce cell cycle arrest and apoptosis, especially cells concurrently harboring *BRAF V600E* mutation and *EZH2* gain.

### Inhibitory effect of combination on PDX models

**Fig. 5:**
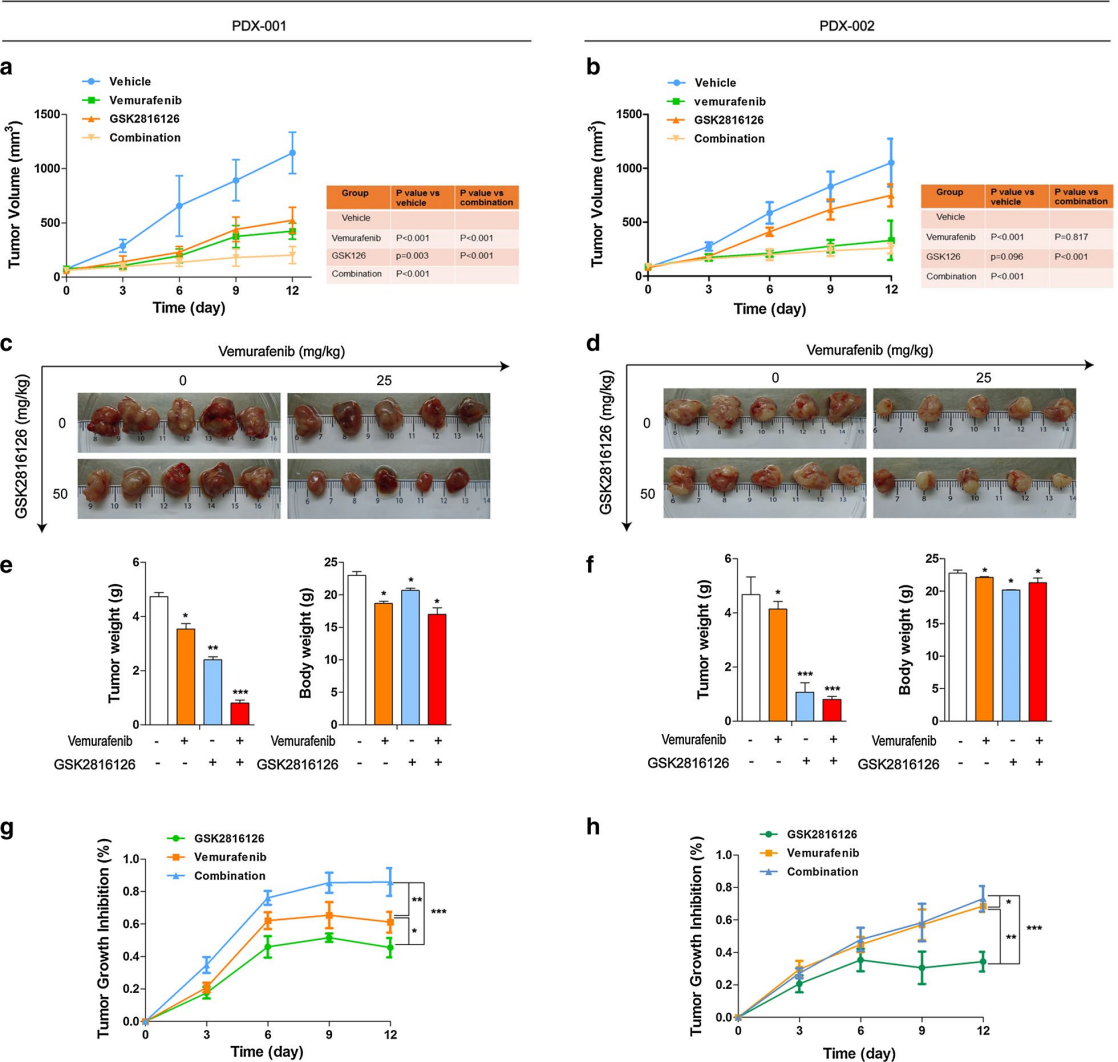
Therapeutic effects of combination of *EZH2* and *BRAF* inhibition in vivo. When the tumor size reached approximately 600 mm^3^, PDX models mice (n = 5) were treated with buffer control, monotherapy and combination therapy daily. Tumor volume was evaluated as [length × width^2^] × 0.5 and presented as mean ± SD. Tumor size (**a**, **b**), tumor volume (**c**, **d**), tumor images (**e**, **f**) tumor weight and body weight (**g**, **h**). Tumor growth inhibition rate. %TGI (tumor growth inhibition) = 100(1 − Wt/Wc); Wt is the mean or median tumor volume of the treated group on day X; Wc is the mean or median tumor volume of controls on day X. The statistical significance of the growth curves (as compared to vehicle group) was evaluated by Mauchly’s test of sphericity (*P < 0.05; **P < 0.01; ***P < 0.001). **a**, **c**, **e**, **g** PDX 001; **b**, **d**, **f**, **h** PDX 002

**Fig. 6:**
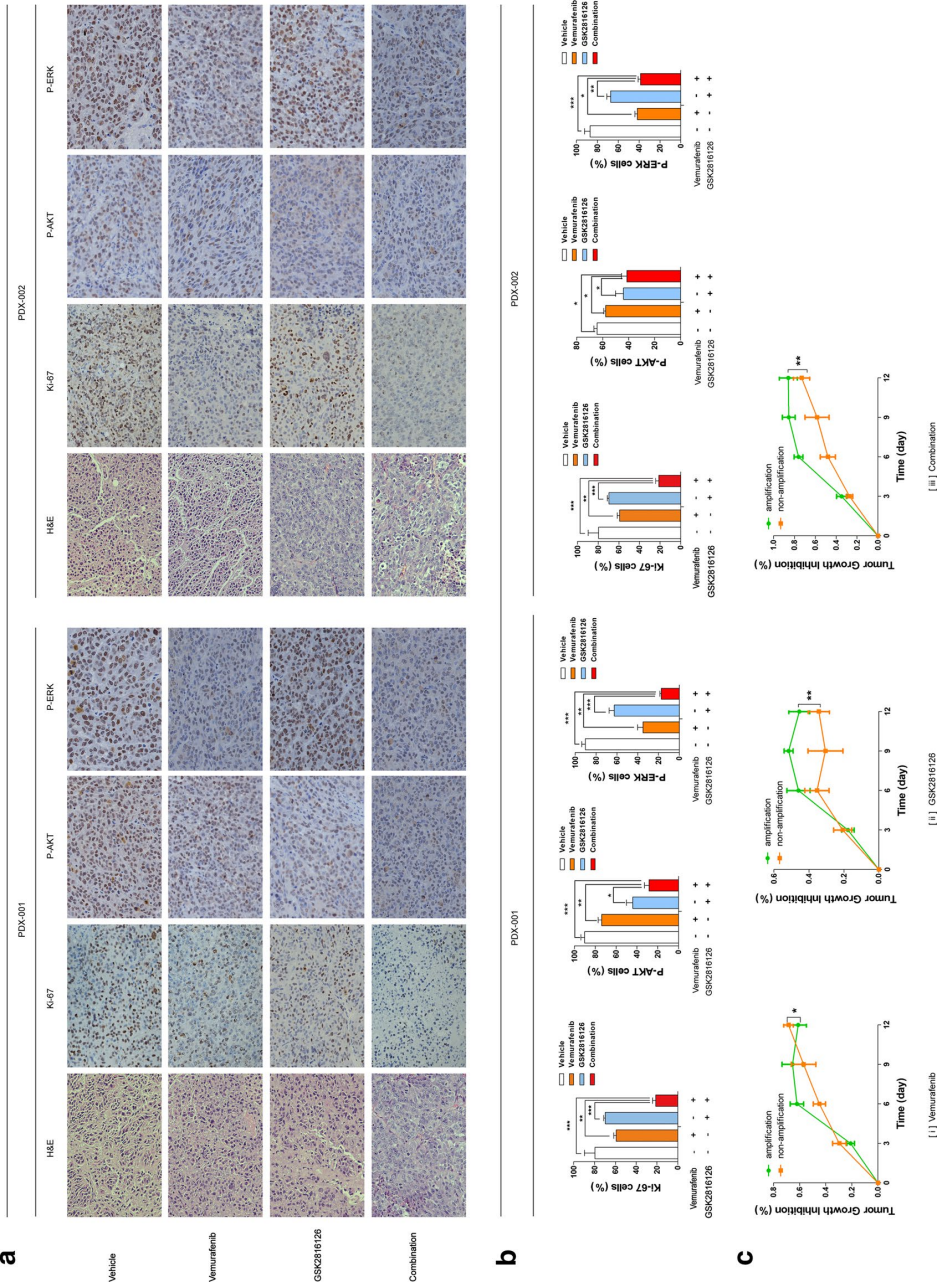
Proliferation index of tumor cells from PDX models after monotherapy and combination therapy. On day 14 of treatments, the tumor nodules were excised and examined by H&E staining and immuohistochemical staining (for Ki-67, P-AKT and P-ERK). The sections were evaluated under microscope, and typical staining was photographed (**a**) and the Ki-67+, P-AKT+ and P-ERK+ cells under five random fields were counted. **b** Graphs of the analysis were shown. The results of were presented as mean ± SD of three sections. The statistical significance was evaluated by One-way ANOVA. (*P < 0.05; **P < 0.01; ***P < 0.001). **c** TGI of three therapeutic regimens on two types of PDX models were shown

To verify the results in cell lines, in vivo assays were performed using PDX mouse models. We found that the combined treatment with vemurafenib and GSK126 significantly inhibited the growth of xenograft tumors in PDX models, especially PDX 001 (concurrently containing *BRAF V600E* and *EZH2* gain) compared with treatment with single drug alone (Fig. 5a, c; *P* < 0.05). In terms of side effects, we did not observe a significant decrease in body weight (Fig. 5e, f). In contrast, in PDX 002 (without *EZH2* gain), the inhibitory efficacy of combined treatment was not significantly different from that of vemurafenib alone but was stronger than single treatment with GSK126 (Fig. 5b, d). Moreover, vemurafenib alone showed stronger inhibitory efficacy than GSK126 alone in two types of PDX mouse models, whereas PDX 001 were more sensitive to GSK126 than PDX 002 models. As further evidence of the inhibitory effects of the three therapeutic regimens on tumor growth, we examined the proliferation of tumor cells in all mice by IHC analysis of Ki-67 (Fig. 6a, b). At last, tumor growth inhibition rate of three therapeutic regimens in same PDX model and each regimen in two types of PDX models was calculated (Figs. 5g, h, 6c). The findings were consistent with the results of changes in tumor volumes. In addition, IHC analysis of P-AKT and P-ERK (Fig. 6a) was also performed, the P-AKT protein levels obviously downregulated in GSK126 monotherapy and combination therapy groups whereas P-ERK protein levels obviously downregulated in vemurafenib monotherapy and combination therapy groups.

These results showed that the combined treatment with vemurafenib and GSK126 inhibited tumor growth of PDX models more efficiently than a single treatment with vemurafenib, especially in those concurrently containing *BRAF V600E* mutation and *EZH2* gain with no obvious toxicity.

## Discussion

Novel therapeutic strategies for the treatment of advanced melanoma is the urgent priority all long, especially *BRAF V600E* mutated melanoma [1]. Even though *BRAF* inhibitor-vemurafenib represented remarkable clinical efficacy, most patients show disease progression within 6–8 months [1].

Recently, progress in combination therapies of melanoma treatment have significantly addressed the limitation of vemurafenib alone, the most successful combination therapeutic regimen was dabrafenib (the second *BRAF* inhibitor approved by FDA) plus trametinib, which induced complete and partial responses in almost 70% of patients and prolonged median progression-free survival in several phase 3 clinical trials, approximately 3–5 months longer than single vemurafenib alone [13]. Therefore, this combination regimen was identified by US NCCN guideline as first-line targeted therapy of advanced melanoma patients containing *BRAF V600E* mutation. However, in many patients with dabrafenib plus trametinib treatment, the disease rapidly progresses and leading to death, such as patients with extremely high LDH concentration [32] and brain metastatic lesions [13], lacking of effective targeted therapies in clinical practice. Therefore, novel combination targets are urgently needed to be found.

Given the high coexistence rate (29%) of *EZH2* gain and *BRAF V600E* mutation in our cohort, we for the first time evaluated the efficacy of combination therapy with *EZH2* and *BRAF* inhibitors in melanoma cell lines and PDX mouse models. We demonstrated that combination therapy was more efficacious than single agent alone in vitro and in vivo, whereas only in melanoma cell lines and PDX models concurrently containing *BRAF V600E* mutation and *EZH2* gain. Therefore, we provided a promising novel therapeutic strategy for melanoma. In patients with *BRAF V600E*-mutated melanoma, the individual *EZH2* gain status should be tested first, and cotreatment with *BRAF* and *EZH2* inhibitors can be applied to patients containing *EZH2* gain and *BRAF V600E* mutation simultaneously to enhance the anticancer effects.

Notably, in our cohort, the frequencies of *EZH2* gain in mucosal (38.9%) and unknown primary melanoma (50%) are markedly higher than acral (21.9%), CSD (25.0%) and non-CSD melanoma (33.3%) subtypes. Therefore, combination therapy with *BRAF* and *EZH2* inhibitors may be particularly beneficial to patients with mucosal and unknown primary melanoma. Moreover, it has been reported that increased activation of the phosphatidylinositol 3-kinase-protein kinase B (PI3K-AKT) signaling pathway has been associated with resistance to *BRAF* and *MEK* inhibitors [32]. Maria E and colleagues found *EZH2* overexpression is sufficient for activation of the PI3K-Akt pathway [33]. According to our western blot and IHC results for P-AKT protein, EZH2 inhibition was significantly associated with downregulation of P-AKT levels. Thus, combination with *EZH2* inhibitors may help to down-regulate the activity of PI3K-AKT pathway and deal with resistance to *BRAF* inhibitor alone and *BRAF* plus *MEK* inhibitors.

We acknowledge several limitations exist in our study. Firstly, the detailed mechanisms involved in the molecular interactions of *BRAF* and *EZH2* gene are still unknown. We speculate that intersection exists between *BRAF* and *EZH2* signaling pathway. Evidence shows that *BRAF* signaling has a role in regulating DNA methylation status, and *EZH2* is a methylation target of *BRAF* [31]. Given the central role of gene methylation in tumorigenesis, *EZH2* and *BRAF* may cooperate to promote the initiation and progression of melanoma [34]. In addition, *BRAF* is the upstream component of the mitogen-activated protein kinase (MAPK) pathway that regulates cell proliferation and survival [35]. *EZH2* is the downstream component of CDK4 pathway that drives cell-cycle progression and regulates cell proliferation [36]. Given the obvious cell-cycle arrest and apoptosis induction was observed after combined treatment, *BRAF* and *EZH2* may cooperate in accelerating cell-cycle progression and inhibiting apoptosis to promote melanoma cell growth. These hypotheses need further study. Secondly, sensitivity of cell lines and PDX models with different *EZH2* gain levels to combined treatment should have been tested to verify whether efficacy of combination therapy is positively correlated with *EZH2* gain levels. In vitro results of our cell lines basically confirmed this idea, however, more cell lines and PDX models with representative, different *EZH2* gain levels should be established to further verify this hypothesis.

Thirdly, although no obvious side effects were observed in our PDX models of combination treatment, further large-scale clinical trials are needed to test the toxicities, potential drug–drug interactions, and optimize concentrations of these two agents in patients with melanoma. Lastly, the effects of other *EZH2* inhibitors, such as EPZ6438 and GSK343, still need to be clarified. All above will be the focus of our subsequent studies.

## Conclusions

In this study, we indicated that coexistence of *BRAF V600E* mutation and *EZH2* gain is rather prevalent in melanoma. Combination with *BRAF* and *EZH2* inhibition showed better inhibitory efficacy compared with vemurafenib monotherapy in vitro and in vivo, especially in melanoma containing concurrently *BRAF V600E* mutation and *EZH2* gain. In conclusion, our study identified *EZH2* gain as a biomarker for selecting patients with *BRAF V600E*-mutated melanoma who may benefit from combination therapy with *BRAF* and *EZH2* inhibitors, thus providing insights into a promising novel therapeutic strategy for the treatment of melanoma.

## Additional files

**Additional file 1.** PDX information.

**Additional file 2.** *EZH2* gain in *BRAF* ^*V600E*^ mutated melanoma. Correlation of *EZH2* gain to clinicopathologic features of *BRAF V600E* mutated melanomas.

**Additional file 3.** Univariate and multivariate analysis of risk factors associated with overall survival.

**Additional file 4.** Compusyn report of combination therapy in A2058 cell line.

**Additional file 5.** Correlation of EZH2 amplification to clinicopathologic features of BRAFV600E mutated mucosal melanomas.

**Additional file 6.** Compusyn report of combination therapy in WM 115 cell line.

**Additional file 7.** Compusyn report of combination therapy in A375 cell line.

**Additional file 8.** Compusyn report of combination therapy in SK-MEL-5 cell line.

**Additional file 9: Figure S1.** Sub-G0 cells detected in 2058 cells after combination therapy. **Figure S2.** Variations in apoptosis rate in all four BRAF V600E mutated cell lines after combination therapy. **Figure S3.** The levels of P-AKT at baseline and after treatment with GSK drug in all cell lines.
