# Supplementary material for: Identification of coexistence of BRAF V600E mutation and EZH2 gain specifically in melanoma as a promising target for combination therapy
Source: J Transl Med. 2017 Dec 4;15:243. doi: 10.1186/s12967-017-1344-z (PMC5716227; doi:10.1186/s12967-017-1344-z)
Supplement: Supplementary file 1 — Additional file 1. PDX information. [file 12967_2017_1344_MOESM1_ESM.docx]

**Additional file 1. PDX information**

|  | Identification number | Gender | Age | Primary | BRAF | NRAS | C-KIT | PDGFR |
| --- | --- | --- | --- | --- | --- | --- | --- | --- |
| PDX001 | T001418142 | man | 50 | acral | V600E | Wild type | Wild type | Wild type |
| PDX002 | T001407187 | woman | 53 | acral | V600E | Wild type | Wild type | Wild type |
