# Supplementary material for: Identification of coexistence of BRAF V600E mutation and EZH2 gain specifically in melanoma as a promising target for combination therapy
Source: J Transl Med. 2017 Dec 4;15:243. doi: 10.1186/s12967-017-1344-z (PMC5716227; doi:10.1186/s12967-017-1344-z)
Supplement: Supplementary file 2 — Additional file 2. EZH2 gain in BRAF V600E mutated melanoma. Correlation of EZH2 gain to clinicopathologic features of BRAF V600E mutated melanomas. [file 12967_2017_1344_MOESM2_ESM.docx]

***Table 1：EZH2 gain in BRAF^V600E^ mutated melanoma***

| **Melanoma subtypes** | **Number**  **of cases** | **Number of case**  **With EZH2 gain（%）** | **Number of case**  **With EZH2 high gain（%）** |
| --- | --- | --- | --- |
| Acral melanoma  Mucosal melanoma  CSD  Non-CSD  Unknown primary  Total  P value | 41  18  36  39  4  138 | 9（21.9）  7（38.9）  9（25.0）  13（33.3）  2（50.0）  40（29.0）  0.346 | 0 (0.0)  1 (20.0)  1 (20.0)  3 (60.0)  0 (0.0)  5 (3.6)  0.500 |

**Table 2：Correlation of *EZH2* gain to clinicopathologic features of *BRAF V600E* mutated melanomas**

|  | | ***EZH2 gain*** | | | |
| --- | --- | --- | --- | --- | --- |
| **Clinicopathologic feature** | | **No gain** | **Gain High gain** | | ***P* value** |
| Age（year）  Gender N（%）  Man  Female  Thickness（mm）  <1  1~2  2~4  >4  Ulceration N（%）  Yes  No  Primary site N（%）  Acral  Mucosal  CSD  Non-CSD  Unknown primary  TNM stage N（%）  Ⅰ  Ⅱ  Ⅲ  Ⅳ | 51.4 ± 13.0  52（53.1）  46（46.9）  0 (0.0)  12 (27.3)  17 (38.6)  15 (34.1)  60（69.0）  27（31.0）  32（32.7）  11（11.2）  27（27.6）  26（26.5）  2（2.0）  3（3.1）  29（29.6）  31（31.6）  35（35.7） | | | 51.5 ± 12.6 50.8 ± 14.0  19（47.5） 2 ( 40.0 )  21（52.5） 3 ( 60.0 )  0 (0.0) 0 (0.0)  3 (27.3) 1 (25.0)  6 (38.6) 1 (25.0)  5 (34.1) 2 (50.0)  12（35.3） 2 (40.0)  22（64.7） 3 (60.0)  9（22.5） 0 (0.0)  7（17.5） 1 (20.0)  9（22.5） 1 (20.0)  13（32.5） 3 (60.0)  2（5.0） 0 (0.0)  4（10.0） 0 (0.0)  5（12.5） 0 (0.0)  17（42.5） 3 (60.0)  14（35.0） 2 (40.0) | 0.960  0.786  0.175  0.012  0.500  0.125 |
