# Supplementary material for: Identification of coexistence of BRAF V600E mutation and EZH2 gain specifically in melanoma as a promising target for combination therapy
Source: J Transl Med. 2017 Dec 4;15:243. doi: 10.1186/s12967-017-1344-z (PMC5716227; doi:10.1186/s12967-017-1344-z)
Supplement: Supplementary file 3 — Additional file 3. Univariate and multivariate analysis of risk factors associated with overall survival. [file 12967_2017_1344_MOESM3_ESM.docx]

| **Variable** | **OS** | | | | | | |
| --- | --- | --- | --- | --- | --- | --- | --- |
|  | **Univariate** | | |  | **Multivariate** | | |
|  | **HR** | **95% CI** | ***P* value** |  | **HR** | **95% CI** | ***P* value** |
| Age (>60 years/≤60 years) | 0.60 | 0.33-1.11 | 0.101 |  |  |  |  |
| Gender (female/male) | 1.14 | 0.67-1.95 | 0.635 |  |  |  |  |
| Ulceration (yes/no) | 0.97 | 0.53-1.79 | 0.764 |  |  |  |  |
| Thickness (≤ 2 mm/>2 mm) | 0.50 | 0.20-1.24 | 0.293 |  |  |  |  |
| TNM stage (I + II/III +IV) | 0.42 | 0.23-0.77 | **0.005** |  | 11.95 | 1.59-89.75 | **0.016** |
| Primary site (AM + MM/CSD + NCSD) | 0.79 | 0.45-1.37 | 0.396 |  |  |  |  |
| *EZH2* gain (yes/no) | 0.50 | 0.02-0.61 | **0.038** |  |  |  |  |
