# Supplementary material for: Identification of coexistence of BRAF V600E mutation and EZH2 gain specifically in melanoma as a promising target for combination therapy
Source: J Transl Med. 2017 Dec 4;15:243. doi: 10.1186/s12967-017-1344-z (PMC5716227; doi:10.1186/s12967-017-1344-z)
Supplement: Supplementary file 4 — Additional file 4. Compusyn report of combination therapy in A2058 cell line. [file 12967_2017_1344_MOESM4_ESM.pdf]

CompuSyn Report

Experiment Name: 2058 GSK126+vemurafenib
Date: 2017/5/6
File Name: C:\Users\»¶»¶\Desktop\BRAF andEZH2\2058lianhejieguo-report\2058lianhejieguo.cse
Description conmbinaton therapy

Drug: GSK126 (5) [uM/L]
Drug: vemurafenib (5) [uM/L]
Drug Combo: combination (4) (5+5 [1:1])

Data for Drug: 5 [uM/L]

Dose	Effect
4.0	0.378
6.0	0.791
8.0	0.941
10.0	0.975
11.0	0.984

5 data points entered.
X-int: 0.64877
Y-int: -2.9679 +/- 0.06428
m: 4.57460 +/- 0.07308
Dm: 4.45421
r: 0.99962

Data for Drug: 5 [uM/L]

Dose	Effect
1.0	0.183
2.0	0.258
3.0	0.473
4.0	0.469
5.0	0.789

5 data points entered.
X-int: 0.49801
Y-int: -0.7717 +/- 0.19802
m: 1.54949 +/- 0.40949
Dm: 3.14782
r: 0.90927

Data for Drug Combo: 4 (5+5 [1:1])

Dose A	Effect
0.8+	0.259
1.0+	0.46
1.2+	0.6
1.6+	0.68
2.0+	0.82

5 data points entered.

X-int: 0.34963
Y-int: -0.9095 +/- 0.12084
m: 2.60144 +/- 0.28570
Dm: 2.23682
r: 0.98239

Dose-Effect Curve

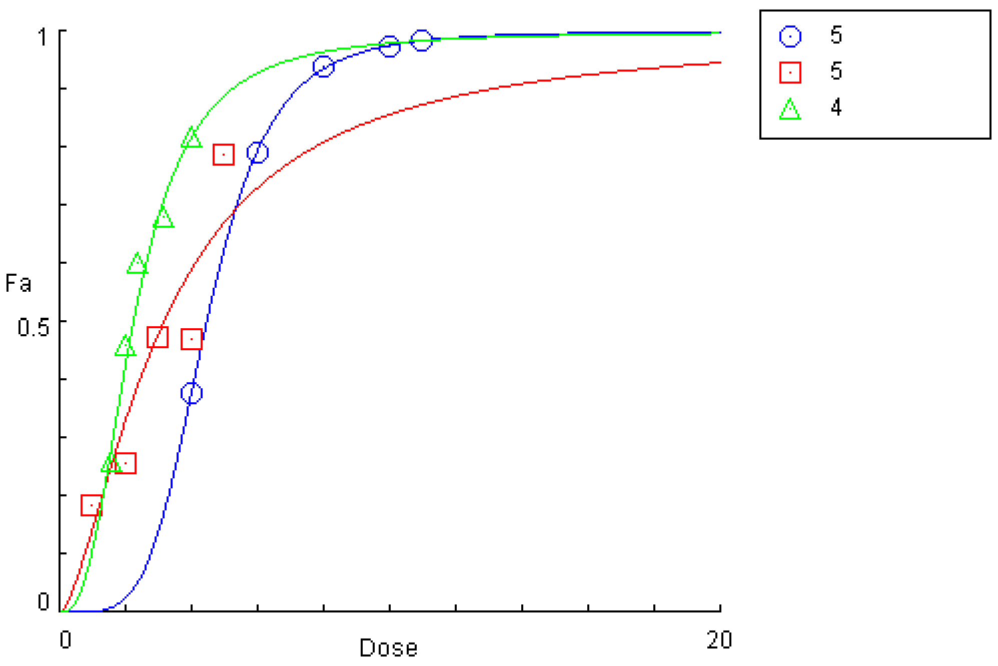

Median-Effect Plot

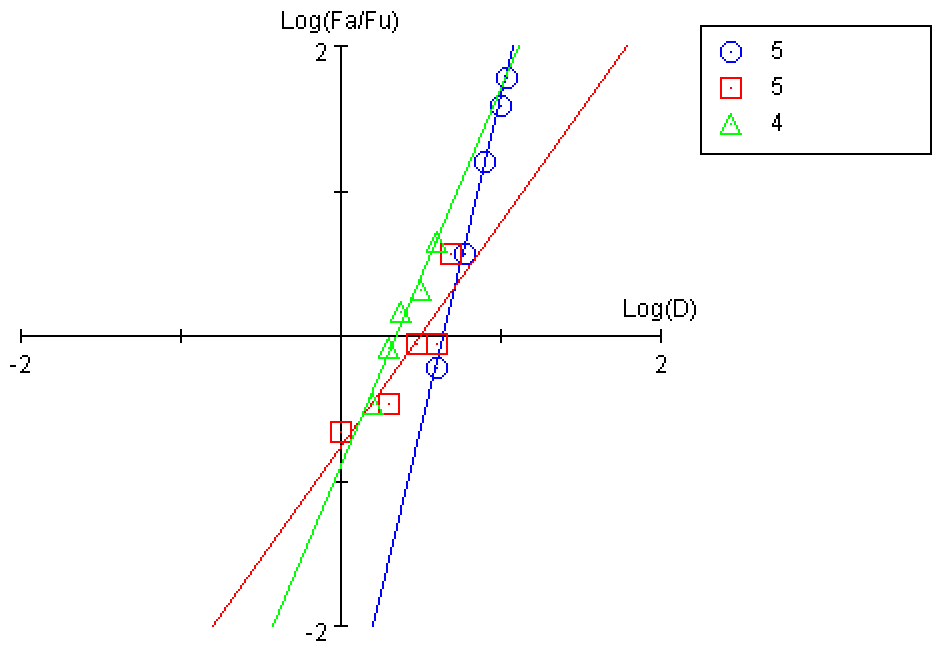

CI Data for Drug Combo: 4 (5+5 [1:1])

Fa	CI Value	Total Dose
0.05	0.92025	0.72123
0.1	0.80484	0.96121
0.15	0.74705	1.14829

0.2	0.70970	1.31280
0.25	0.68255	1.46631
0.3	0.66141	1.61503
0.35	0.64419	1.76314
0.4	0.62972	1.91400
0.45	0.61727	2.07076
0.5	0.60639	2.23682
0.55	0.59675	2.41619
0.6	0.58817	2.61409
0.65	0.58053	2.83776
0.7	0.57378	3.09800
0.75	0.56799	3.41222
0.8	0.56342	3.81122
0.85	0.56070	4.35724
0.9	0.56169	5.20527
0.95	0.57389	6.93723
0.97	0.59024	8.51027

CI values for actual experimental points:

**Total Dose**	**Fa**	**CI Value**
1.6	0.259	0.72685
2.0	0.46	0.58483
2.4	0.6	0.54000
3.2	0.68	0.61713
4.0	0.82	0.56112

Combination Index Plot

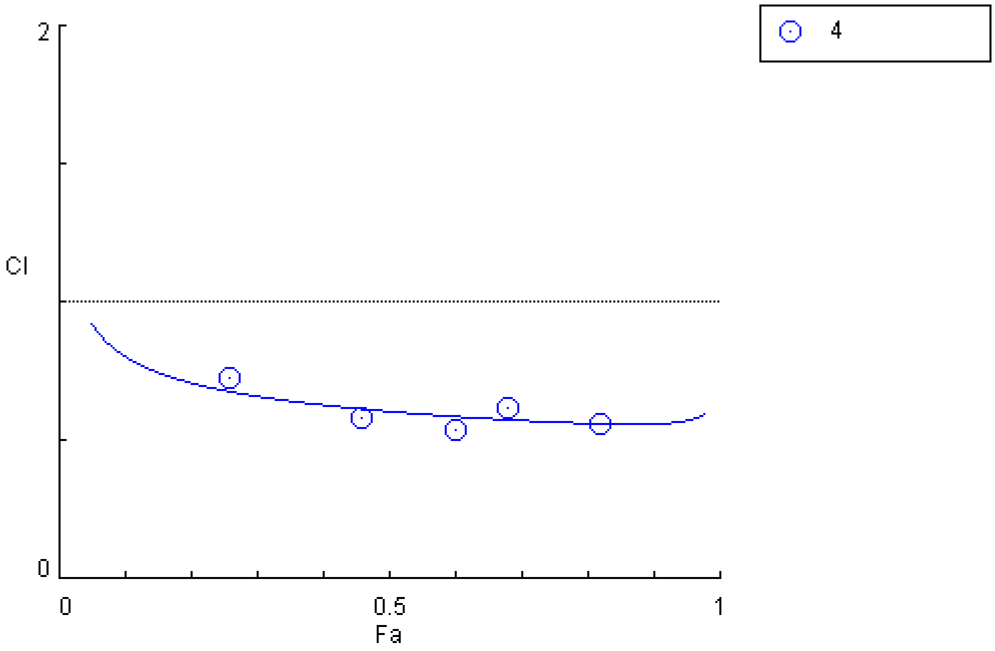

Logarithmic Combination Index Plot

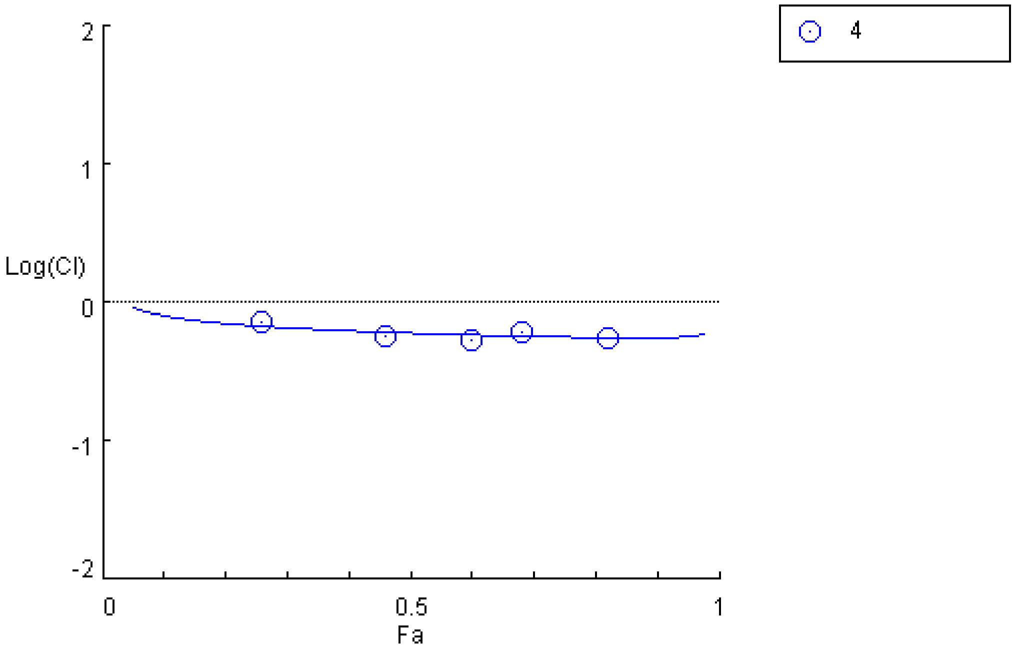

DRI Data for Drug Combo: 4 (5+5 [1:1])

Fa	Dose 5	Dose 5	DRI 5	DRI 5
0.05	2.34011	0.47069	6.48920	1.30523
0.1	2.75534	0.76237	5.73306	1.58627
0.15	3.04856	1.02761	5.30975	1.78982
0.2	3.28974	1.28663	5.01180	1.96014
0.25	3.50326	1.54913	4.77835	2.11297
0.3	3.70111	1.82191	4.58334	2.25619
0.35	3.89047	2.11107	4.41312	2.39468
0.4	4.07641	2.42306	4.25958	2.53194
0.45	4.26304	2.76544	4.11737	2.67094
0.5	4.45421	3.14782	3.98263	2.81455
0.55	4.65395	3.58306	3.85230	2.96587
0.6	4.86703	4.08935	3.72369	3.12870
0.65	5.09964	4.69371	3.59414	3.30804
0.7	5.36056	5.43867	3.46065	3.51108
0.75	5.66329	6.39635	3.31942	3.74908
0.8	6.03087	7.70131	3.16480	4.04139
0.85	6.50799	9.64248	2.98721	4.42596
0.9	7.20057	12.9973	2.76664	4.99391
0.95	8.47821	21.0516	2.44427	6.06918
0.97	9.52309	29.6689	2.23802	6.97249

DRI values calculated at experimental points

Fa	Dose 5	Dose 5	DRI 5	DRI 5
0.259	3.53978	1.59729	4.42473	1.99661
0.46	4.30079	2.83837	4.30079	2.83837
0.6	4.86703	4.08935	4.05586	3.40779
0.68	5.25207	5.12011	3.28255	3.20007
0.82	6.20479	8.37560	3.10239	4.18780

DRI Plot for Combo: 4 (5+5 [1:1])

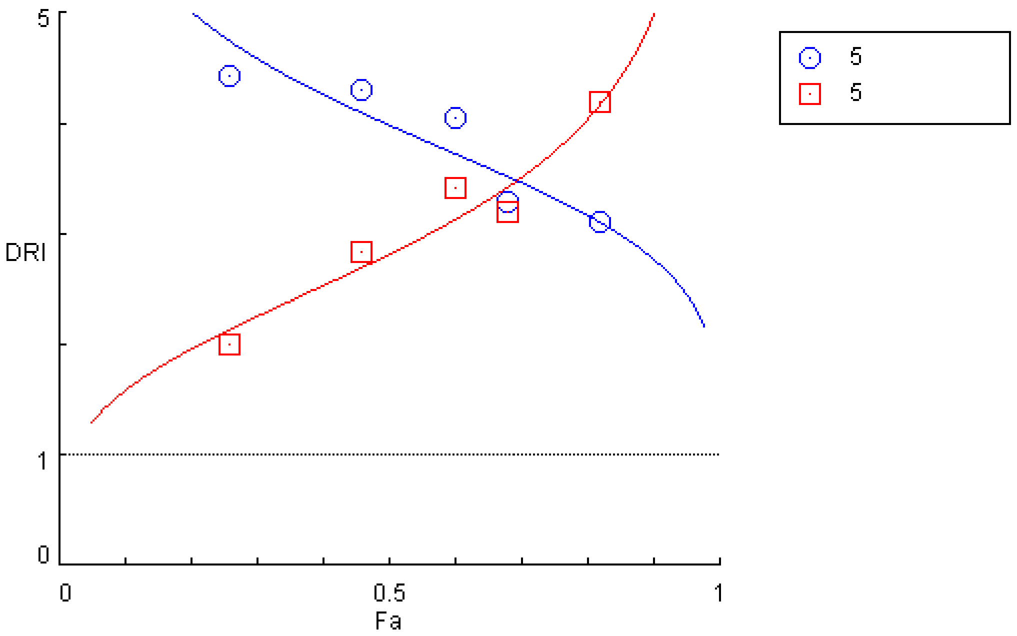

Log(DRI) Plot for Combo: 4 (5+5 [1:1])

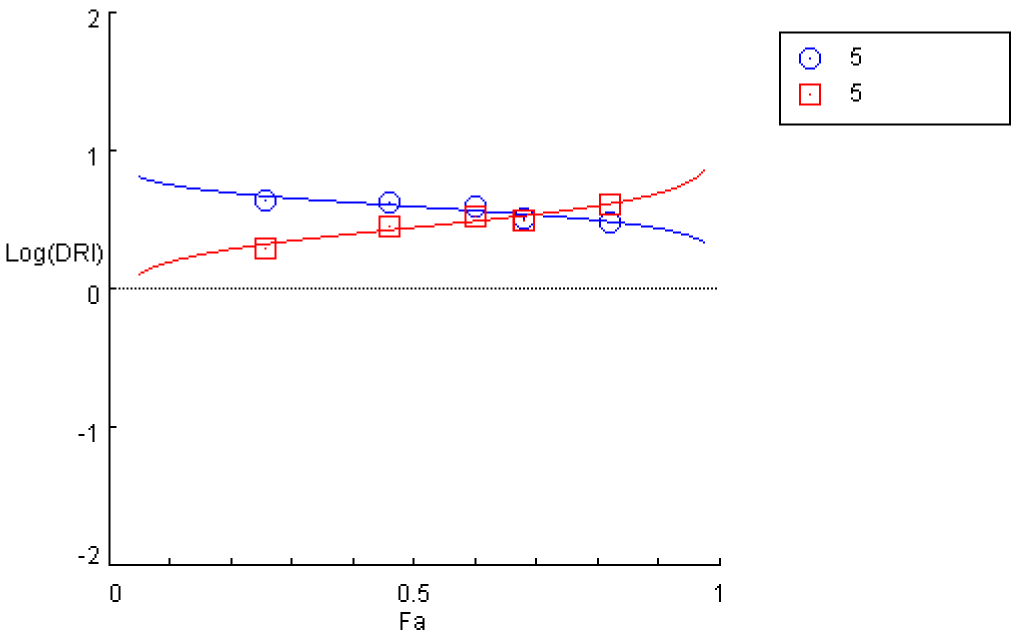

Isobologram for Combo: 4 (5+5 [1:1])

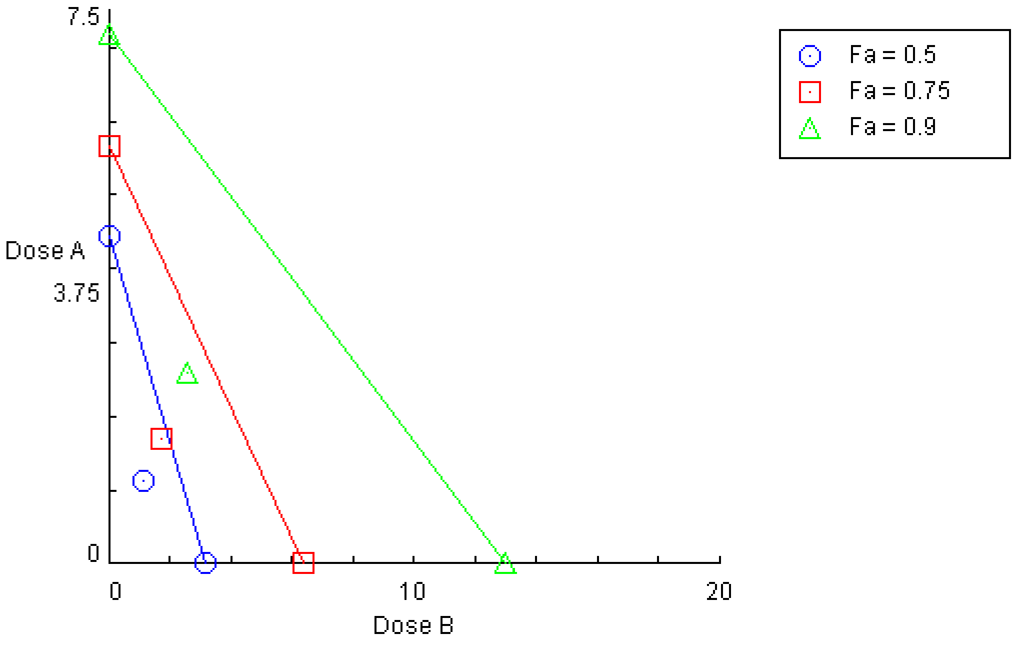

Polygonogram at Fa = 0.9

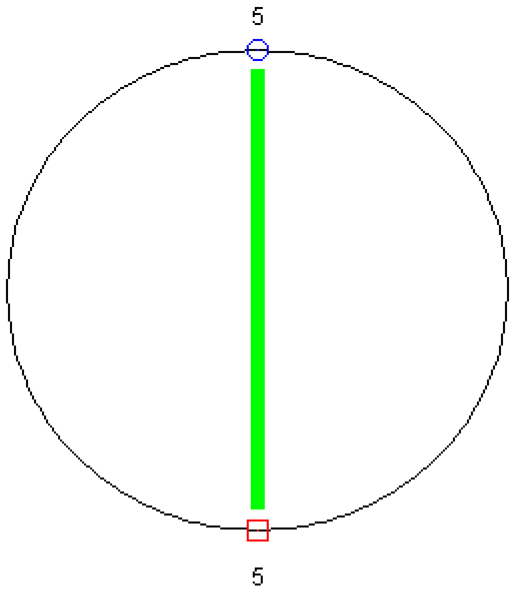

Summary Table

Experiment Name: 2058 GSK126+vemurafenib
Date: 2017/5/6
File Name: C:\Users\»¶»¶\Desktop\BRAF andEZH2\2058lianhejieguo-report\2058lianhejieguo.cse
Description conmbinaton therapy

Drug: GSK126 (5) [uM/L]
Drug: vemurafenib (5) [uM/L]
Drug Combo: combination (4) (5+5 [1:1])

Drug/Combo	Dm	m	r
5	4.45421	4.57460	0.99962
5	3.14782	1.54949	0.90927
4	2.23682	2.60144	0.98239

CI values at:

Combo	ED50	ED75	ED90	ED95
4	0.60639	0.56799	0.56169	0.57389

Data for Fa = 0.5

Drug/Combo	CI value	Dose 5	Dose 5
5		4.45421	
5			3.14782
4	0.60639	1.11841	1.11841

Data for Fa = 0.75

Drug/Combo	CI value	Dose 5	Dose 5
5		5.66329	
5			6.39635
4	0.56799	1.70611	1.70611

Data for Fa = 0.9

Drug/Combo	CI value	Dose 5	Dose 5
5		7.20057	
5			12.9973
4	0.56169	2.60264	2.60264

Data for Fa = 0.95

Drug/Combo	CI value	Dose 5	Dose 5
5		8.47821	
5			21.0516
4	0.57389	3.46861	3.46861

Data for Fa = 0.97

Drug/Combo	CI value	Dose 5	Dose 5
5		9.52309	
5			29.6689
4	0.59024	4.25513	4.25513
