# Supplementary material for: Identification of coexistence of BRAF V600E mutation and EZH2 gain specifically in melanoma as a promising target for combination therapy
Source: J Transl Med. 2017 Dec 4;15:243. doi: 10.1186/s12967-017-1344-z (PMC5716227; doi:10.1186/s12967-017-1344-z)
Supplement: Supplementary file 5 — Additional file 5. Correlation of EZH2 amplification to clinicopathologic features of BRAFV600E mutated mucosal melanomas. [file 12967_2017_1344_MOESM5_ESM.docx]

**Additional File 5**：***Correlation of EZH2 amplification to clinicopathologic features of BRAF^V600E^ mutated mucosal melanomas***

|  | | ***EZH2 genotype*** | | | |
| --- | --- | --- | --- | --- | --- |
| **Clinicopathologic feature** | | **gain** | | **No gain** | ***P* value** |
| Age（year）  Gender N（%）  Man  Female  Thickness（mm）  <1  1~2  2~4  >4  Ulceration N（%）  Yes  No  TNM stage N（%）  Ⅰ  Ⅱ  Ⅲ  Ⅳ | 52.7 ± 11.6  6（54.5）  5（45.5）  0(0.0)  0(0.0)  1(100.0)  0(0.0)  2（33.3）  4（66.7）  0（0.0）  2（28.6）  1（14.3）  4（57.1） | | 53.1 ± 11.8  1（16.7）  5（83.3）  0(0.0)  0(0.0)  0(0.0)  2(100.0)  8（80.0）  2（20.0）  0（0.0）  7（63.6）  1（9.1）  3（27.3） | | 0.639  0.786  0.175  0.009  0.125 |

^a^ For evaluation of age, the unpaired t or t' tests were used. For evaluation of gender, ulceration and stages, the chi-square tests or Fisher's exact tests were used. For evaluation of thickness, Mann-Whitney U tests were used.
