# Supplementary material for: Identification of coexistence of BRAF V600E mutation and EZH2 gain specifically in melanoma as a promising target for combination therapy
Source: J Transl Med. 2017 Dec 4;15:243. doi: 10.1186/s12967-017-1344-z (PMC5716227; doi:10.1186/s12967-017-1344-z)
Supplement: Supplementary file 6 — Additional file 6. Compusyn report of combination therapy in WM 115 cell line. [file 12967_2017_1344_MOESM6_ESM.pdf]

CompuSyn Report

Experiment Name: 1675
Date: 2017-5-6
File Name: C:\Users\»¶»¶\Desktop\BRAF andEZH2\1675lianhejieguo-report\1675ZUIHOU.cse
Description combination

Drug: GSK126 (6) [uM/L]
Drug: vemurafenib (5) [uM/L]
Drug Combo: conbination (5) (6+5 [5:1])

Data for Drug: 6 [uM/L]

Dose	Effect
4.0	0.001
6.0	0.014
8.0	0.067
10.0	0.249
11.0	0.634
12.0	0.911

6 data points entered.
X-int: 1.01284
Y-int: -7.8565 +/- 0.80436
m: 7.75683 +/- 0.87826
Dm: 10.3002
r: 0.97531

Data for Drug: 5 [uM/L]

Dose	Effect
0.6	0.222
0.8	0.355
1.0	0.561
1.2	0.579
1.6	0.612

5 data points entered.
X-int: 0.03233
Y-int: -0.0592 +/- 0.05816
m: 1.83225 +/- 0.39841
Dm: 1.07728
r: 0.93583

Data for Drug Combo: 5 (6+5 [5:1])

Dose A	Effect
4.0+	0.222
6.0+	0.452
8.0+	0.501
10.0+	0.541
11.0+	0.597

5 data points entered.

X-int: 0.99564

Y-int: -1.4860 +/- 0.24147

m: 1.49253 +/- 0.25218

Dm: 9.90012

r: 0.95975

Dose-Effect Curve

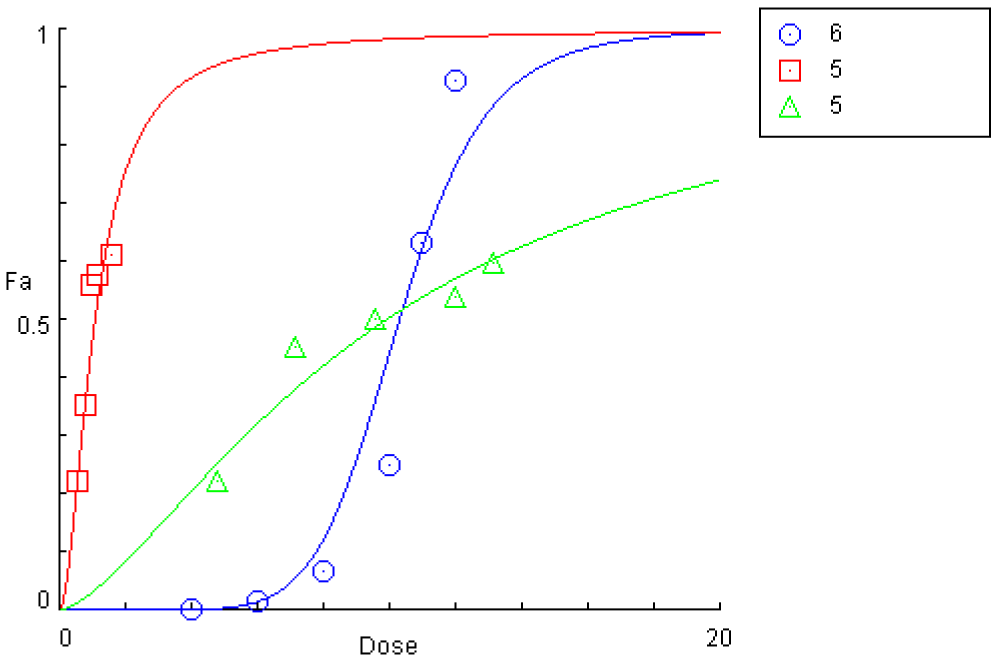

Median-Effect Plot

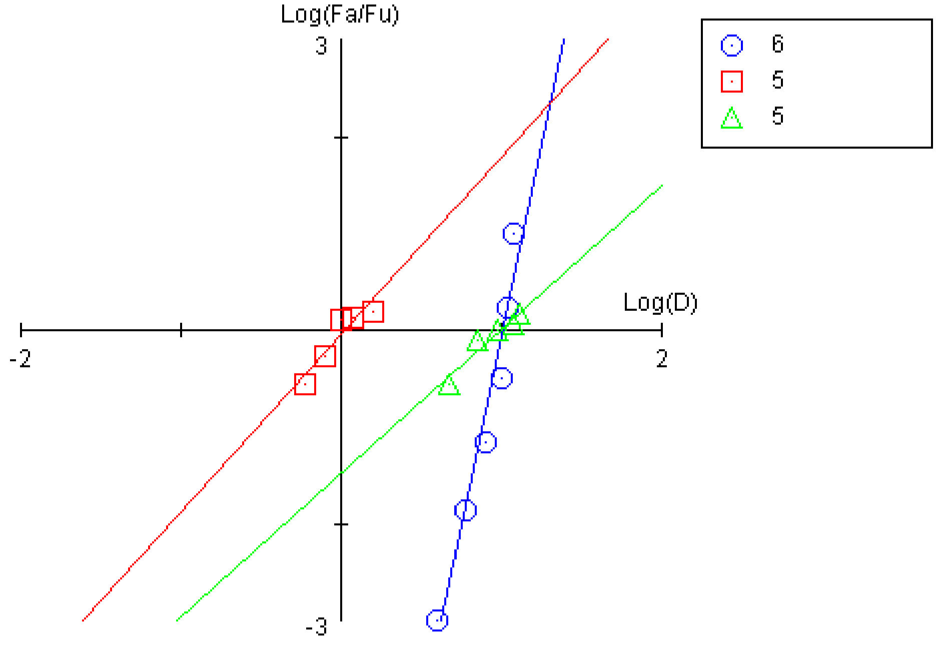

CI Data for Drug Combo: 5 (6+5 [5:1])

Fa	CI Value	Total Dose
0.05	1.22525	1.37679
0.1	1.40972	2.27140

0.15	1.54806	3.09675
0.2	1.66765	3.91072
0.25	1.77828	4.74206
0.3	1.88504	5.61169
0.35	1.99127	6.53902
0.4	2.09959	7.54498
0.45	2.21249	8.65465
0.5	2.33262	9.90012
0.55	2.46315	11.3248
0.6	2.60824	12.9904
0.65	2.77374	14.9888
0.7	2.96851	17.4657
0.75	3.20700	20.6688
0.8	3.51534	25.0625
0.85	3.94750	31.6500
0.9	4.64228	43.1507
0.95	6.14843	71.1889
0.97	7.61259	101.652

CI values for actual experimental points:

**Total Dose**	**Fa**	**CI Value**
4.8	0.222	1.92882
7.2	0.452	1.83454
9.6	0.501	2.25827
12.0	0.541	2.64773
13.2	0.597	2.66315

Combination Index Plot

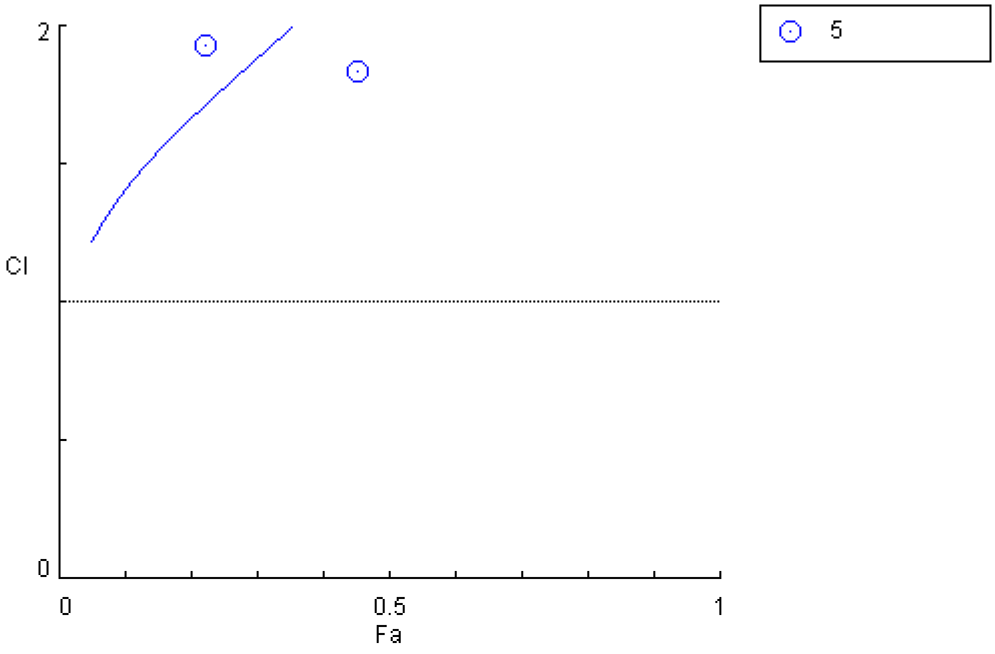

Logarithmic Combination Index Plot

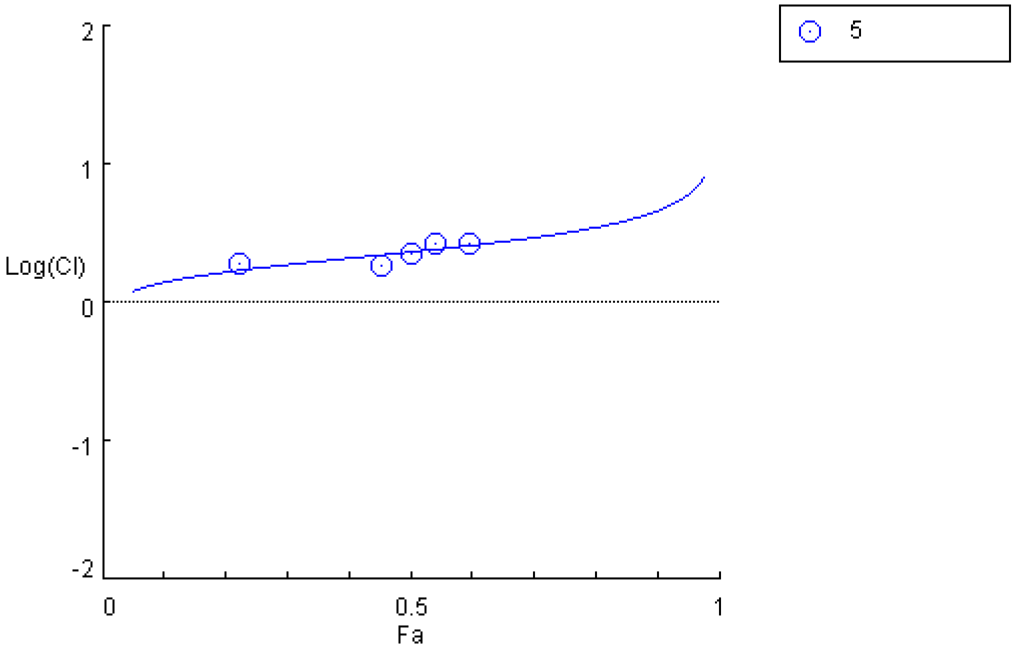

DRI Data for Drug Combo: 5 (6+5 [5:1])

Fa	Dose 6	Dose 5	DRI 6	DRI 5
0.05	7.04675	0.21598	6.14189	0.94124
0.1	7.75934	0.32473	4.09933	0.85780
0.15	8.23619	0.41800	3.19154	0.80989
0.2	8.61445	0.50552	2.64333	0.77559
0.25	8.93994	0.59146	2.26229	0.74836
0.3	9.23433	0.67842	1.97466	0.72536
0.35	9.51010	0.76842	1.74523	0.70508
0.4	9.77559	0.86342	1.55477	0.68662
0.45	10.0371	0.96553	1.39168	0.66937
0.5	10.3002	1.07728	1.24849	0.65289
0.55	10.5701	1.20197	1.12003	0.63682
0.6	10.8529	1.34411	1.00255	0.62082
0.65	11.1559	1.51028	0.89313	0.60456
0.7	11.4890	1.71065	0.78936	0.58766
0.75	11.8674	1.96214	0.68900	0.56960
0.8	12.3158	2.29572	0.58968	0.54960
0.85	12.8814	2.77637	0.48839	0.52633
0.9	13.6730	3.57381	0.38024	0.49693
0.95	15.0556	5.37331	0.25379	0.45288
0.97	16.1238	7.18224	0.19034	0.42393

DRI values calculated at experimental points

Fa	Dose 6	Dose 5	DRI 6	DRI 5
0.222	8.76258	0.54336	2.19064	0.67920
0.452	10.0476	0.96979	1.67460	0.80816
0.501	10.3055	1.07964	1.28818	0.67477
0.541	10.5208	1.17839	1.05208	0.58920
0.597	10.8354	1.33498	0.98504	0.60681

DRI Plot for Combo: 5 (6+5 [5:1])

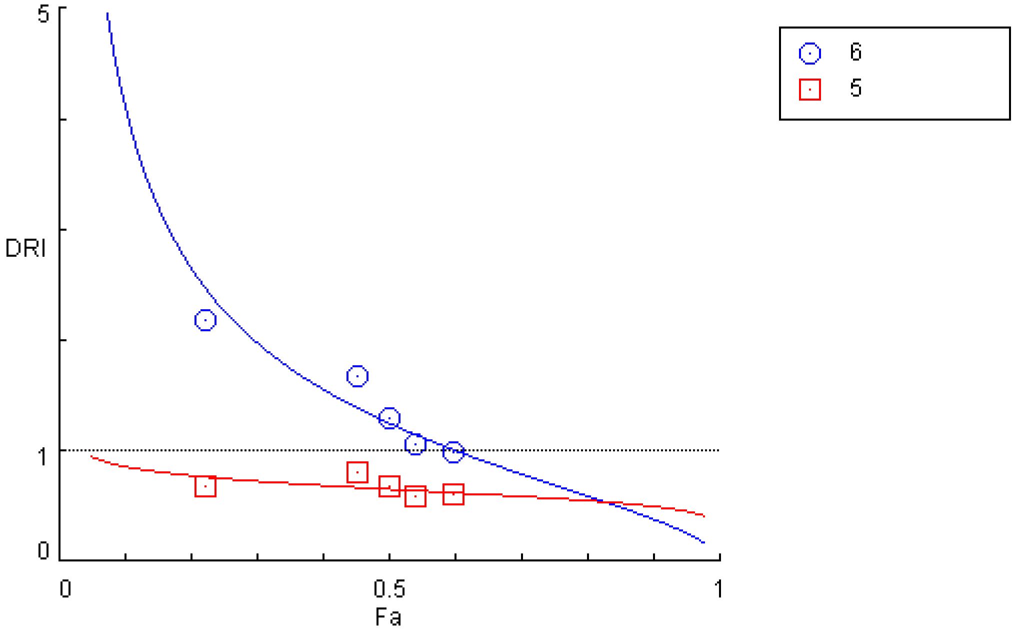

Log(DRI) Plot for Combo: 5 (6+5 [5:1])

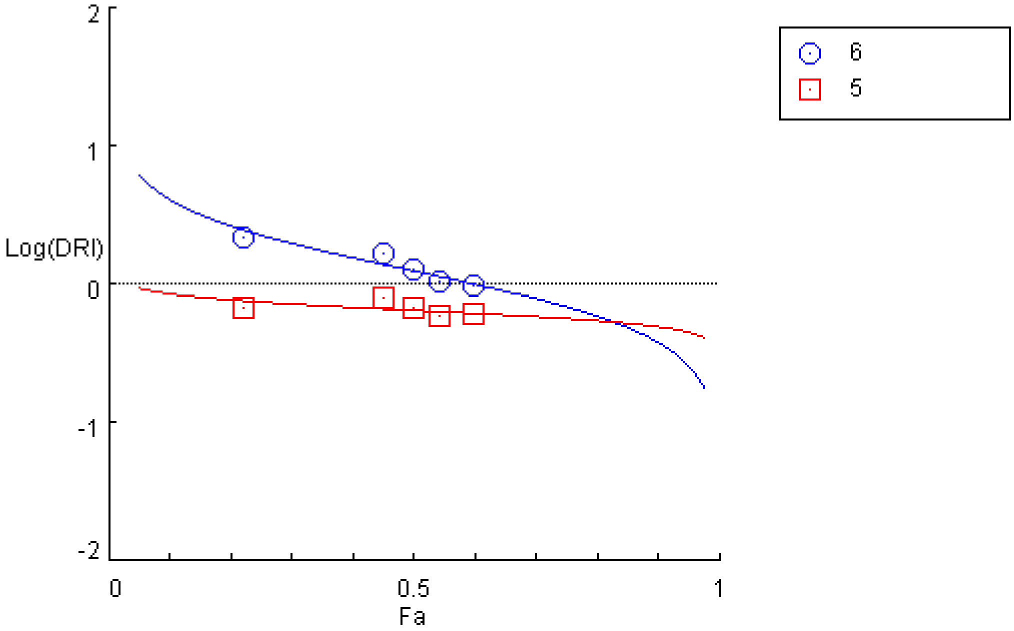

Isobologram for Combo: 5 (6+5 [5:1])

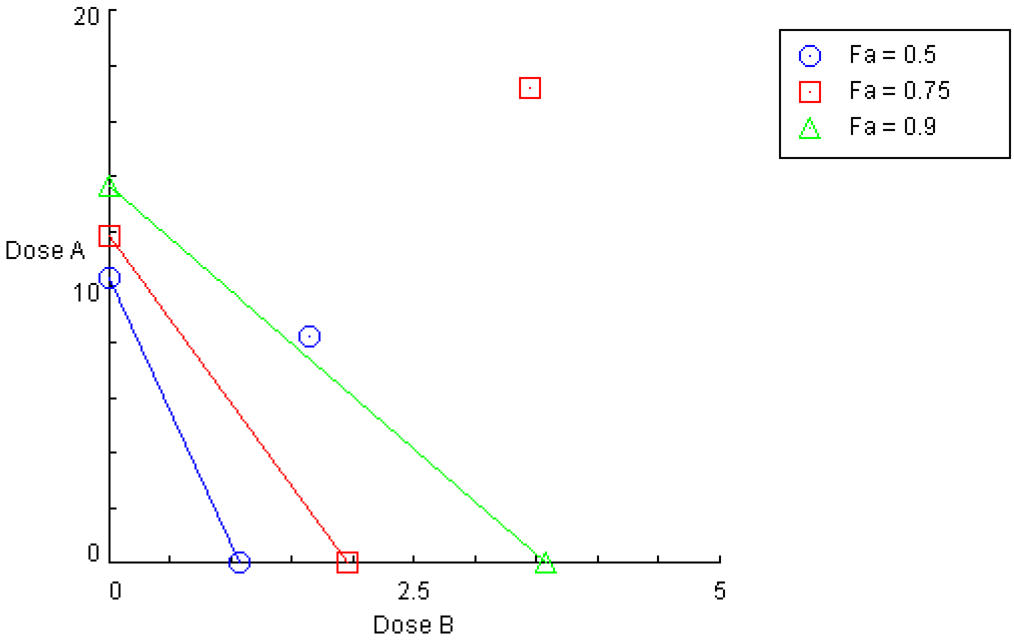

Polygonogram at Fa = 0.9

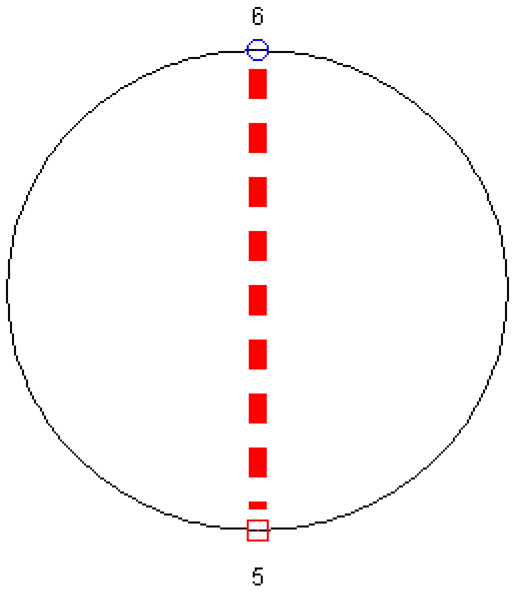

Summary Table

**Experiment Name:**	1675
**Date:**	2017-5-6
**File Name:**	C:\Users\»¶»¶\Desktop\BRAF andEZH2\1675lianhejieguo-report\1675ZUIHOU.cse
**Description**	combination

**Drug:**	GSK126 (6) [uM/L]
**Drug:**	vemurafenib (5) [uM/L]
**Drug Combo:**	conbination (5) (6+5 [5:1])

Drug/Combo	Dm	m	r
6	10.3002	7.75683	0.97531
5	1.07728	1.83225	0.93583
5	9.90012	1.49253	0.95975

CI values at:

Combo	ED50	ED75	ED90	ED95
5	2.33262	3.20700	4.64228	6.14843

Data for Fa = 0.5

Drug/Combo	CI value	Dose 6	Dose 5
6		10.3002	
5			1.07728
5	2.33262	8.25010	1.65002

Data for Fa = 0.75

Drug/Combo	CI value	Dose 6	Dose 5
6		11.8674	
5			1.96214
5	3.20700	17.2240	3.44479

Data for Fa = 0.9

Drug/Combo	CI value	Dose 6	Dose 5
6		13.6730	
5			3.57381
5	4.64228	35.9589	7.19179

Data for Fa = 0.95

Drug/Combo	CI value	Dose 6	Dose 5
6		15.0556	
5			5.37331
5	6.14843	59.3241	11.8648

Data for Fa = 0.97

Drug/Combo	CI value	Dose 6	Dose 5
6		16.1238	
5			7.18224
5	7.61259	84.7097	16.9419
