# Supplementary material for: Identification of coexistence of BRAF V600E mutation and EZH2 gain specifically in melanoma as a promising target for combination therapy
Source: J Transl Med. 2017 Dec 4;15:243. doi: 10.1186/s12967-017-1344-z (PMC5716227; doi:10.1186/s12967-017-1344-z)
Supplement: Supplementary file 7 — Additional file 7. Compusyn report of combination therapy in A375 cell line. [file 12967_2017_1344_MOESM7_ESM.pdf]

CompuSyn Report

Experiment Name: 375combination
Date: 2017/9/13
File Name: C:\Users\»¶»¶\Desktop\375.cse
Description

Drug: vemurafenib (6) [uM/L]
Drug: GSK126 (6) [uM/L]
Drug Combo: combination (5) (6+6 [1:12])

Data for Drug: 6 [uM/L]

Dose	Effect
0.6	0.046
0.8	0.079
1.0	0.232
1.2	0.36
1.6	0.618
2.0	0.963

6 data points entered.
X-int: 0.09606
Y-int: -0.4726 +/- 0.12886
m: 4.91949 +/- 0.71085
Dm: 1.24755
r: 0.96069

Data for Drug: 6 [uM/L]

Dose	Effect
2.0	0.055
4.0	0.0949
6.0	0.1682
8.0	0.2226
10.0	0.4087
12.0	0.7033

6 data points entered.
X-int: 1.06809
Y-int: -1.9815 +/- 0.32241
m: 1.85520 +/- 0.39297
Dm: 11.6975
r: 0.92078

Data for Drug Combo: 5 (6+6 [1:12])

Dose A	Effect
0.4+	0.3796
0.6+	0.5361
0.8+	0.6949
1.0+	0.7718

1.2+ 0.8888
5 data points entered.
X-int: 0.83955
Y-int: -1.8734 +/- 0.25740
m: 2.23147 +/- 0.25722
Dm: 6.91122
r: 0.98065

Dose-Effect Curve

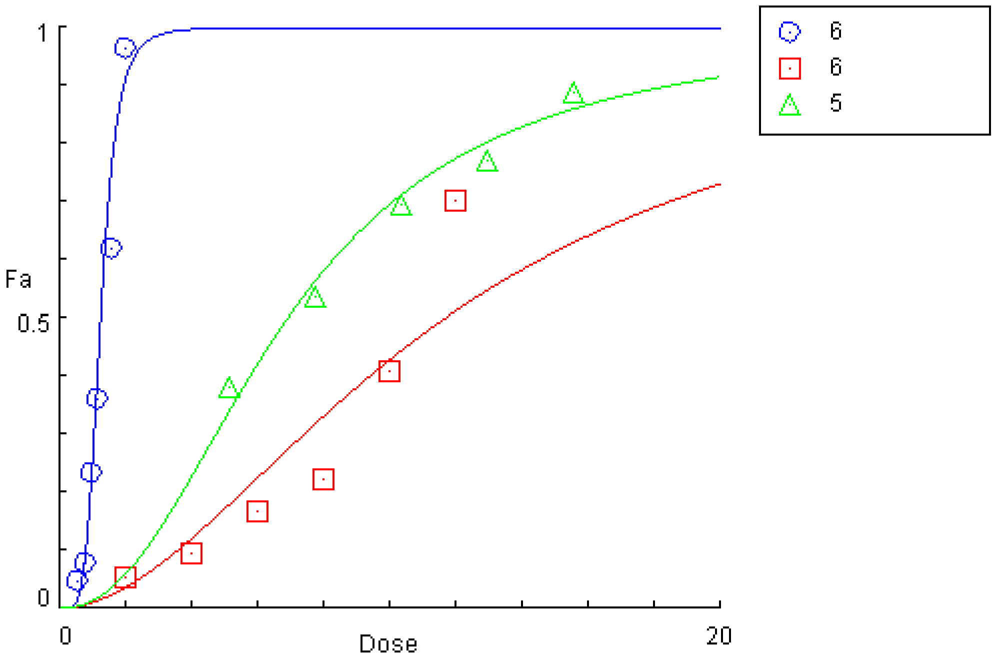

Median-Effect Plot

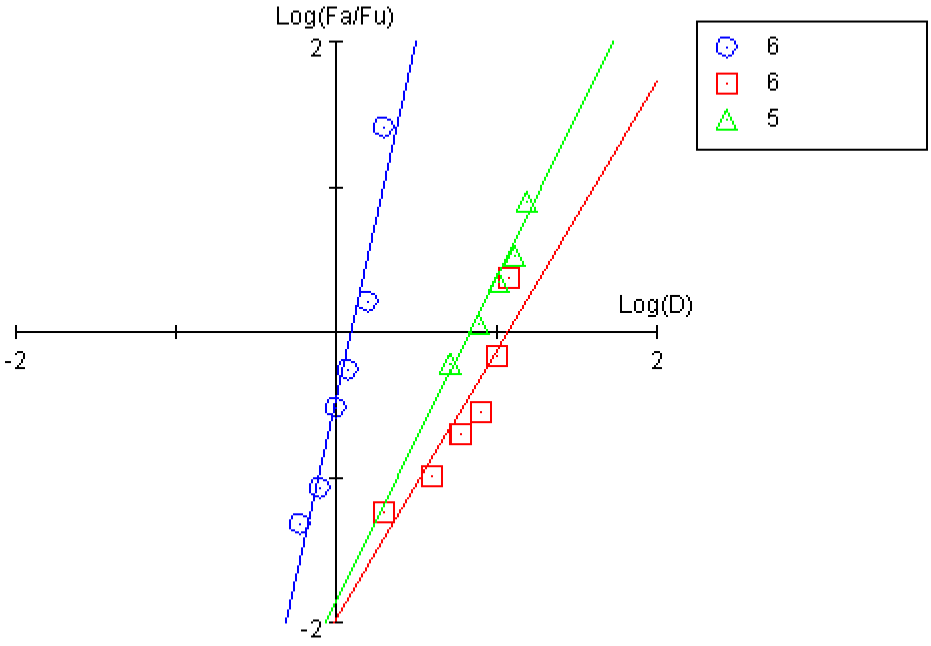

CI Data for Drug Combo: 5 (6+6 [1:12])

Fa	CI Value	Total Dose
0.05	0.91995	1.84714

0.1	0.91476	2.58181
0.15	0.91719	3.17660
0.2	0.92210	3.71322
0.25	0.92828	4.22416
0.3	0.93534	4.72772
0.35	0.94315	5.23692
0.4	0.95172	5.76291
0.45	0.96113	6.31684
0.5	0.97153	6.91122
0.55	0.98313	7.56154
0.6	0.99627	8.28834
0.65	1.01143	9.12082
0.7	1.02935	10.1032
0.75	1.05123	11.3076
0.8	1.07920	12.8635
0.85	1.11749	15.0365
0.9	1.17647	18.5006
0.95	1.29367	25.8589
0.97	1.39583	32.8158

CI values for actual experimental points:

**Total Dose**	**Fa**	**CI Value**
5.2	0.3796	0.88905
7.8	0.5361	1.03636
10.4	0.6949	1.06907
13.0	0.7718	1.15763
15.6	0.8888	1.03193

Combination Index Plot

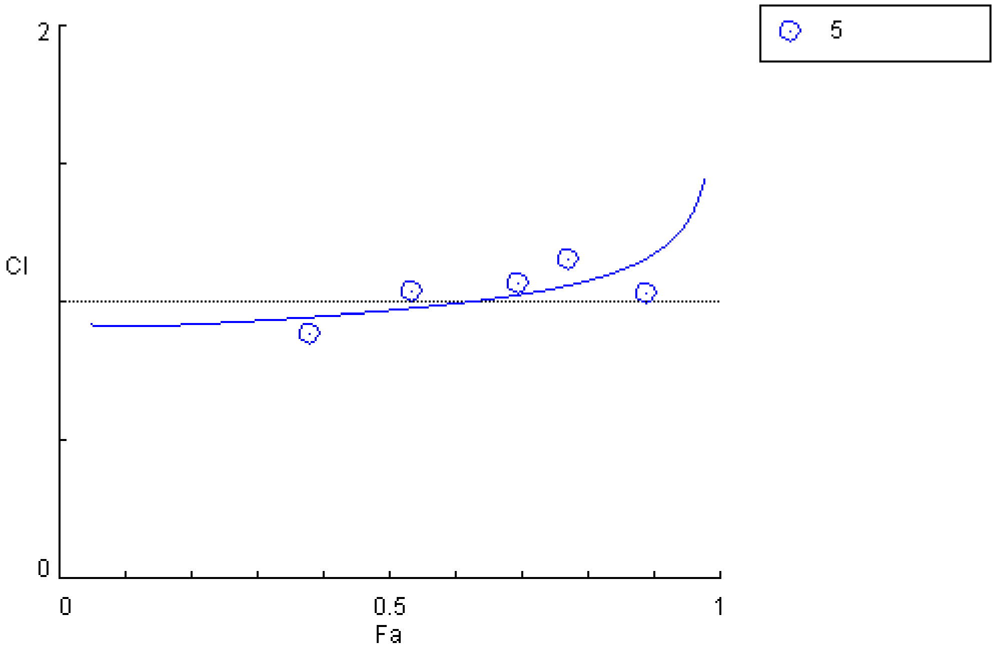

Logarithmic Combination Index Plot

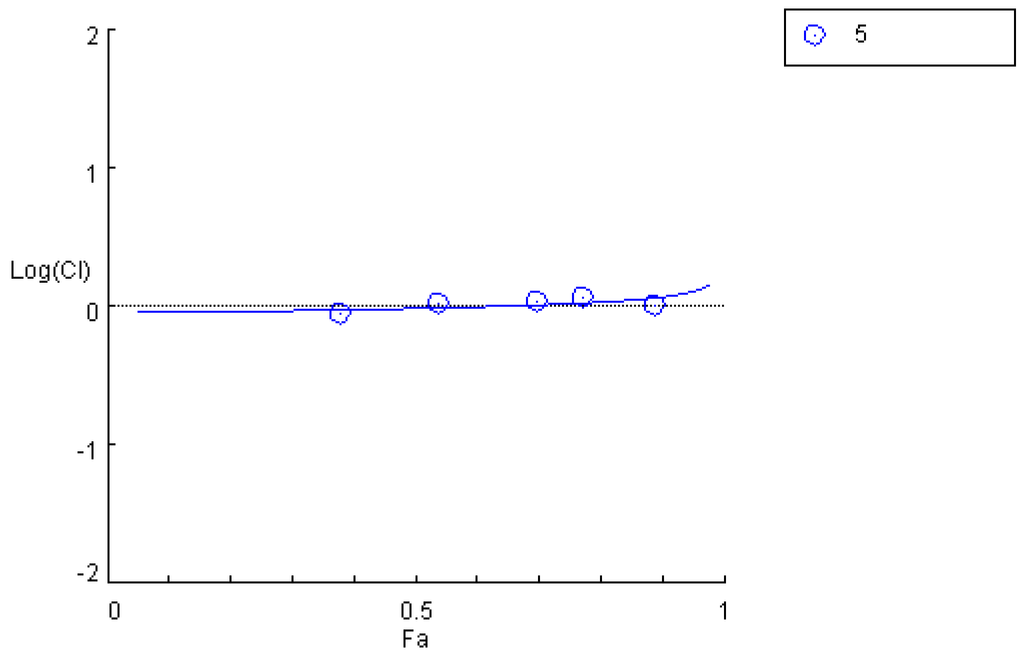

DRI Data for Drug Combo: 5 (6+6 [1:12])

Fa	Dose 6	Dose 6	DRI 6	DRI 6
0.05	0.68568	2.39227	4.82574	1.40305
0.1	0.79815	3.57874	4.01887	1.50165
0.15	0.87685	4.59228	3.58845	1.56613
0.2	0.94118	5.54072	3.29509	1.61651
0.25	0.99786	6.47010	3.07096	1.65933
0.3	1.05016	7.40871	2.88768	1.69767
0.35	1.10004	8.37871	2.73071	1.73326
0.4	1.14885	9.40099	2.59157	1.76723
0.45	1.19768	10.4982	2.46482	1.80043
0.5	1.24755	11.6975	2.34663	1.83357
0.55	1.29949	13.0337	2.23411	1.86732
0.6	1.35473	14.5549	2.12484	1.90241
0.65	1.41484	16.3307	2.01658	1.93970
0.7	1.48203	18.4689	1.90696	1.98036
0.75	1.55971	21.1481	1.79315	2.02611
0.8	1.65363	24.6954	1.67118	2.07979
0.85	1.77496	29.7957	1.53456	2.14668
0.9	1.94997	38.2342	1.37021	2.23887
0.95	2.26983	57.1968	1.14111	2.39620
0.97	2.52888	76.1782	1.00182	2.51484

DRI values calculated at experimental points

Fa	Dose 6	Dose 6	DRI 6	DRI 6
0.3796	1.12899	8.97620	2.82247	1.87004
0.5361	1.28477	12.6460	2.14129	1.75639
0.6949	1.47477	18.2298	1.84346	1.89894
0.7718	1.59818	22.5599	1.59818	1.87999
0.8888	1.90349	35.8648	1.58624	2.49061

DRI Plot for Combo: 5 (6+6 [1:12])

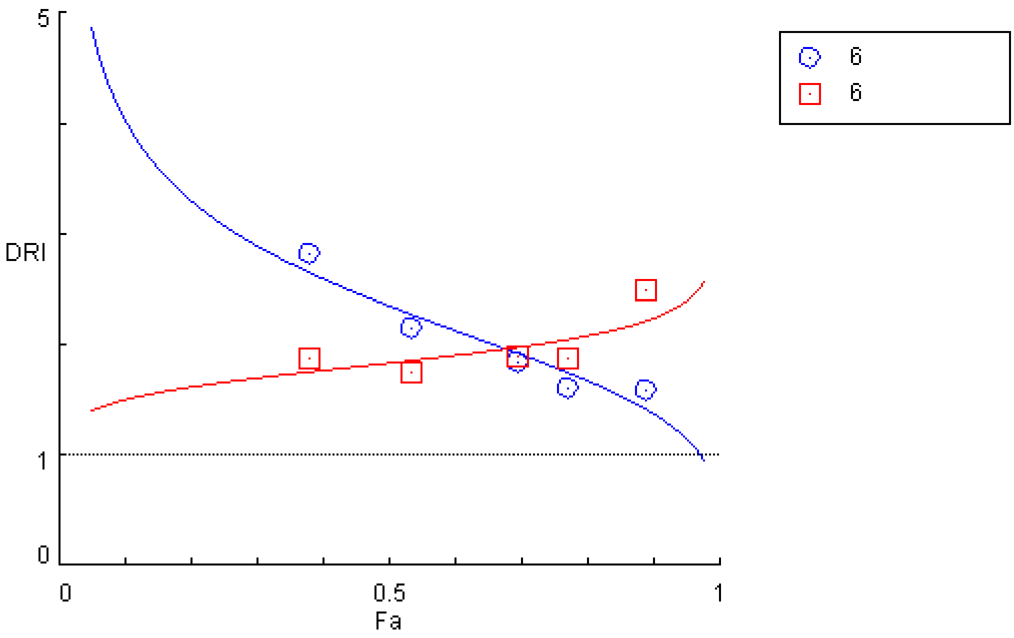

Log(DRI) Plot for Combo: 5 (6+6 [1:12])

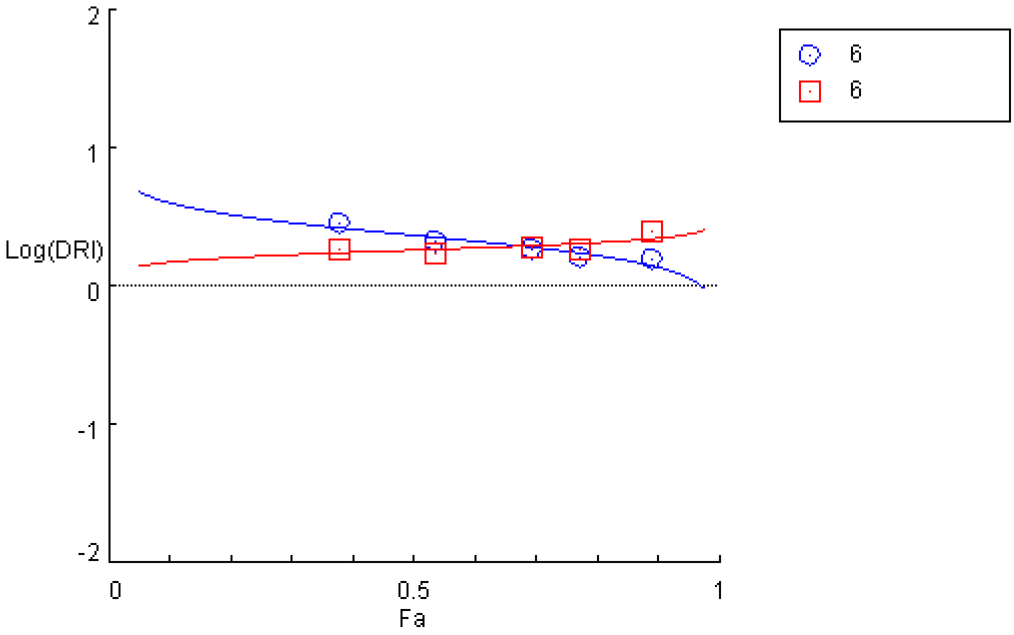

Isobologram for Combo: 5 (6+6 [1:12])

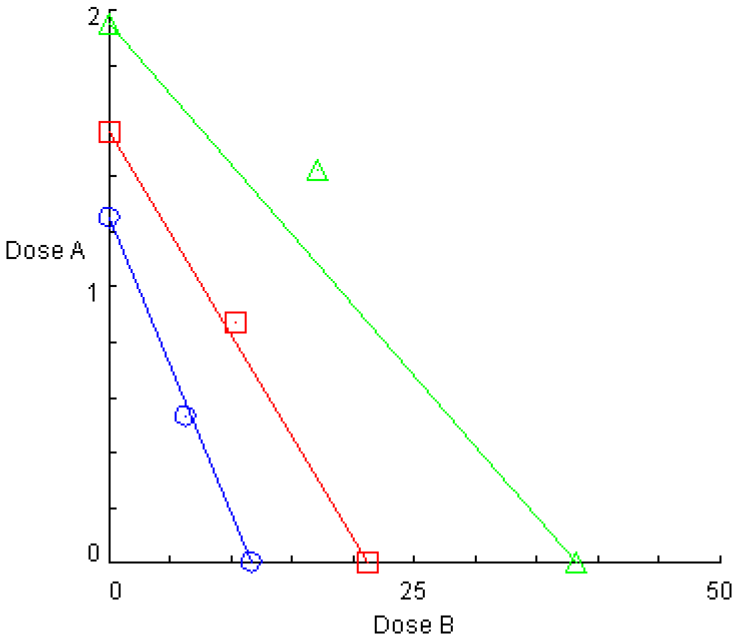

Polygonogram at Fa = 0.9

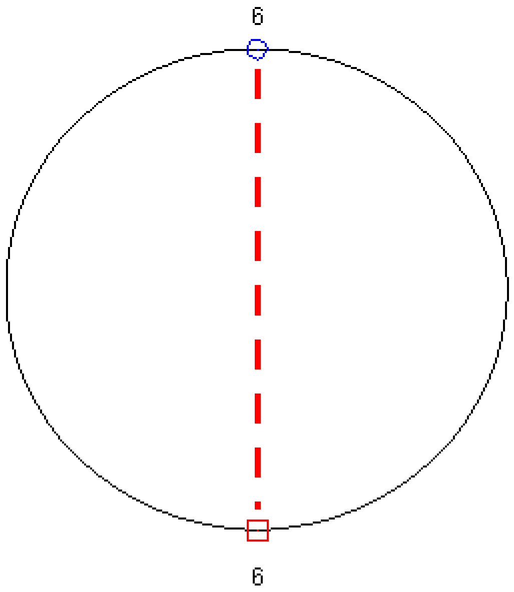

Summary Table

**Experiment Name:**	375combination
**Date:**	2017/9/13
**File Name:**	C:\Users\»¶»¶\Desktop\375.cse
**Description**	

Drug: vemurafenib (6) [uM/L]
Drug: GSK126 (6) [uM/L]
Drug Combo: combination (5) (6+6 [1:12])

Drug/Combo	Dm	m	r
6	1.24755	4.91949	0.96069
6	11.6975	1.85520	0.92078
5	6.91122	2.23147	0.98065

CI values at:				
**Combo**	**ED50**	**ED75**	**ED90**	**ED95**
5	0.97153	1.05123	1.17647	1.29367

Data for Fa = 0.5

**Drug/Combo**	**CI value**	**Dose 6**	**Dose 6**
6		1.24755	
6			11.6975
5	0.97153	0.53163	6.37959

Data for Fa = 0.75

**Drug/Combo**	**CI value**	**Dose 6**	**Dose 6**
6		1.55971	
6			21.1481
5	1.05123	0.86981	10.4378

Data for Fa = 0.9

**Drug/Combo**	**CI value**	**Dose 6**	**Dose 6**
6		1.94997	
6			38.2342
5	1.17647	1.42312	17.0774

Data for Fa = 0.95

**Drug/Combo**	**CI value**	**Dose 6**	**Dose 6**
6		2.26983	
6			57.1968
5	1.29367	1.98915	23.8697

Data for Fa = 0.97

**Drug/Combo**	**CI value**	**Dose 6**	**Dose 6**
6		2.52888	
6			76.1782
5	1.39583	2.52429	30.2915
