# Supplementary material for: Identification of coexistence of BRAF V600E mutation and EZH2 gain specifically in melanoma as a promising target for combination therapy
Source: J Transl Med. 2017 Dec 4;15:243. doi: 10.1186/s12967-017-1344-z (PMC5716227; doi:10.1186/s12967-017-1344-z)
Supplement: Supplementary file 8 — Additional file 8. Compusyn report of combination therapy in SK-MEL-5 cell line. [file 12967_2017_1344_MOESM8_ESM.pdf]

CompuSyn Report

**Experiment Name:**	SK5-LIANHE
**Date:**	2017/9/13
**File Name:**	C:\Users\»¶»¶\Desktop\SK-5.cse
**Description**	COMBINATION

Drug: vemurafenib (6) [uM/L]
Drug: GSK126 (6) [uM/L]
Drug Combo: combination (6) (6+6 [1:7])

Data for Drug: 6 [uM/L]

Dose	Effect
0.6	0.068
0.8	0.18
1.0	0.189
1.2	0.232
1.6	0.446
2.0	0.653

6 data points entered.

X-int: 0.22985
Y-int: -0.5713 +/- 0.05119
m: 2.48537 +/- 0.28240
Dm: 1.69764
r: 0.97514

Data for Drug: 6 [uM/L]

Dose	Effect
2.0	0.09
4.0	0.338
6.0	0.416
8.0	0.585
10.0	0.741
12.0	0.882

6 data points entered.

X-int: 0.77460
Y-int: -1.7092 +/- 0.19536
m: 2.20659 +/- 0.23811
Dm: 5.95110
r: 0.97749

Data for Drug Combo: 6 (6+6 [1:7])

Dose A	Effect
0.6+	0.439
0.8+	0.526
1.0+	0.717
1.2+	0.8599

1.6+ 0.9497
2.0+ 0.9773
6 data points entered.
X-int: 0.75678
Y-int: -2.6741 +/- 0.27323
m: 3.53355 +/- 0.28358
Dm: 5.71183
r: 0.98736

Dose-Effect Curve

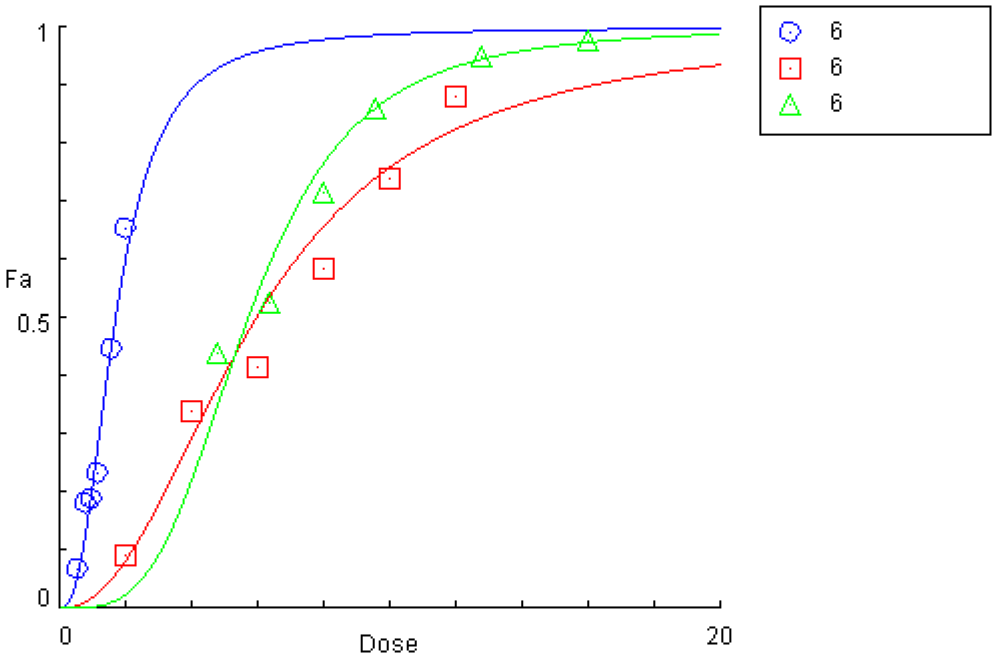

Median-Effect Plot

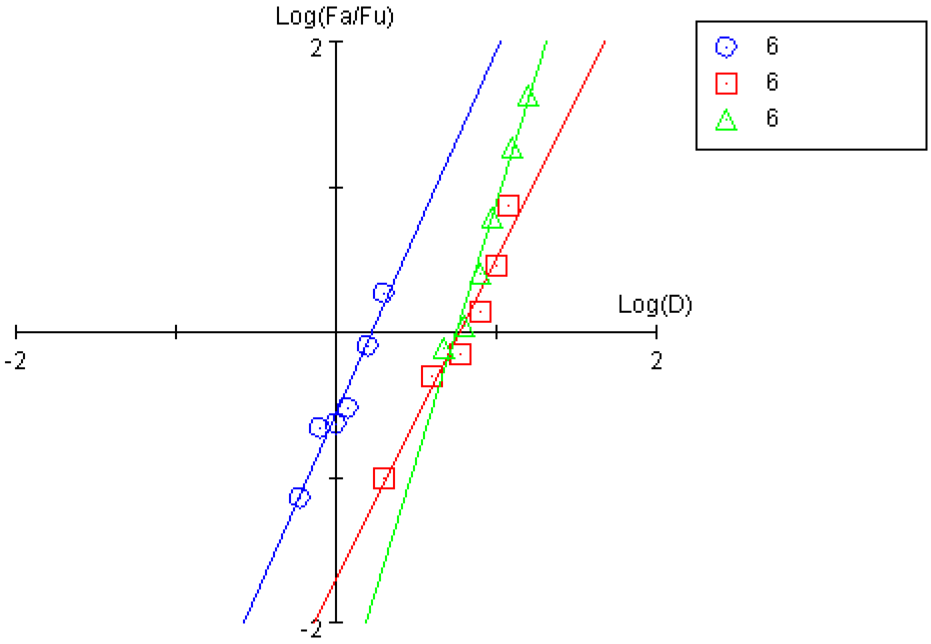

CI Data for Drug Combo: 6 (6+6 [1:7])

Fa	CI Value	Total Dose

0.05	1.98383	2.48248
0.1	1.76731	3.06707
0.15	1.64553	3.49609
0.2	1.55953	3.85826
0.25	1.49198	4.18552
0.3	1.43542	4.49404
0.35	1.38595	4.79393
0.4	1.34124	5.09262
0.45	1.29976	5.39649
0.5	1.26039	5.71183
0.55	1.22224	6.04559
0.6	1.18453	6.40633
0.65	1.14646	6.80548
0.7	1.10716	7.25961
0.75	1.06549	7.79474
0.8	1.01976	8.45589
0.85	0.96707	9.33186
0.9	0.90137	10.6372
0.95	0.80476	13.1421
0.97	0.74255	15.2760

CI values for actual experimental points:

**Total Dose**	**Fa**	**CI Value**
4.8	0.439	1.17879
6.4	0.526	1.34956
8.0	0.717	1.17709
9.6	0.8599	0.96087
12.8	0.9497	0.78596
16.0	0.9773	0.68684

Combination Index Plot

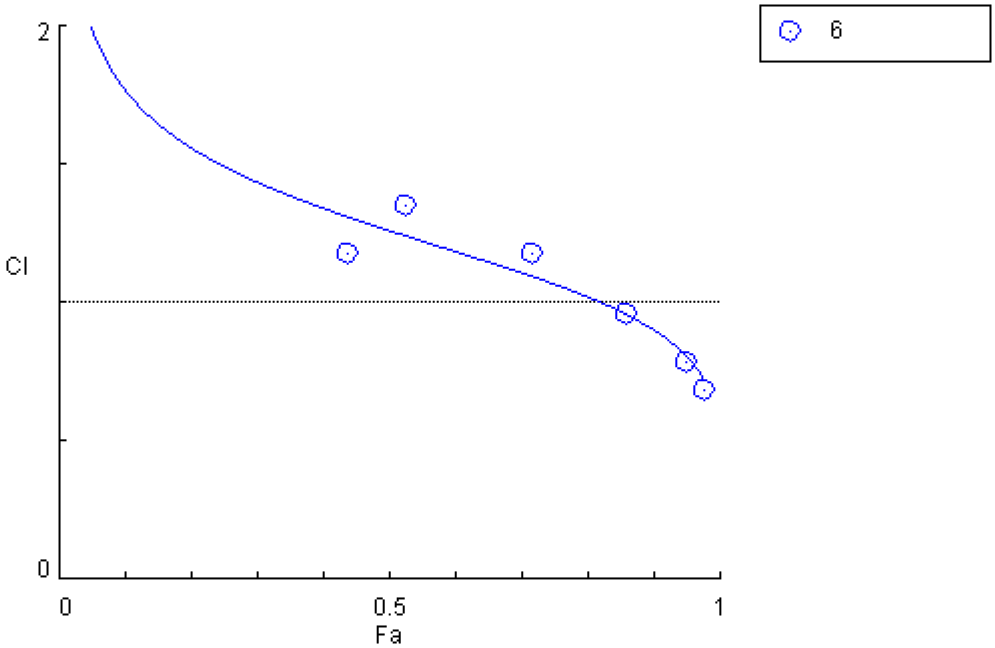

Logarithmic Combination Index Plot

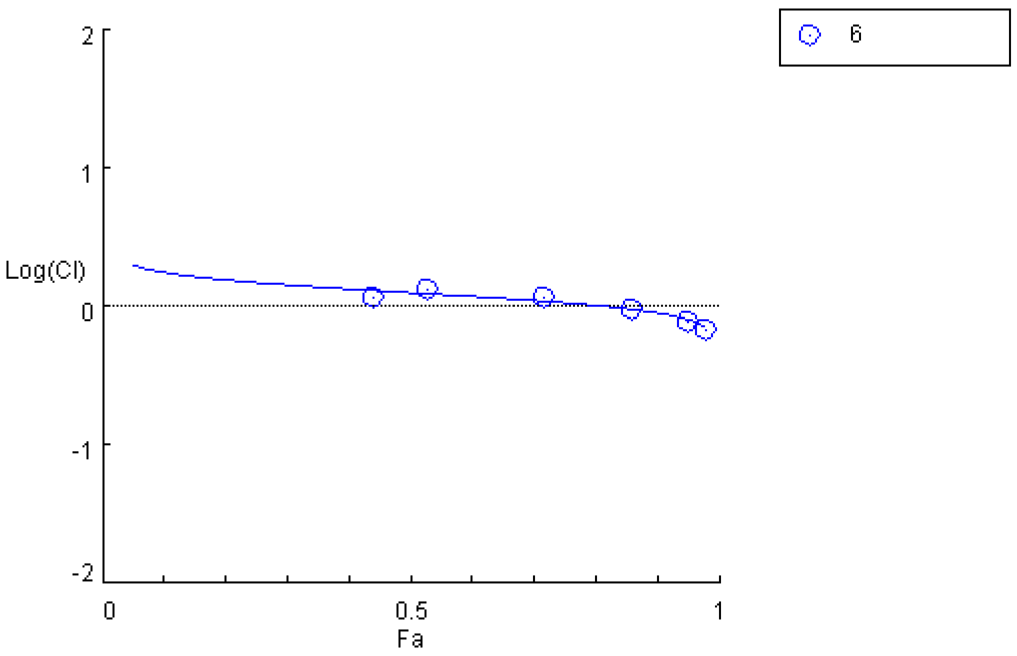

DRI Data for Drug Combo: 6 (6+6 [1:7])

Fa	Dose 6	Dose 6	DRI 6	DRI 6
0.05	0.51920	1.56704	1.67316	0.72142
0.1	0.70130	2.19860	1.82923	0.81925
0.15	0.84478	2.71142	1.93308	0.88635
0.2	0.97186	3.17505	2.01513	0.94048
0.25	1.09112	3.61719	2.08552	0.98767
0.3	1.20723	4.05354	2.14903	1.03083
0.35	1.32335	4.49531	2.20838	1.07167
0.4	1.44210	4.95216	2.26539	1.11134
0.45	1.56596	5.43377	2.32145	1.15075
0.5	1.69764	5.95110	2.37772	1.19073
0.55	1.84040	6.51767	2.43536	1.23210
0.6	1.99847	7.15154	2.49562	1.27580
0.65	2.17780	7.87834	2.56005	1.32302
0.7	2.38728	8.73695	2.63075	1.37543
0.75	2.64130	9.79090	2.71086	1.43553
0.8	2.96543	11.1543	2.80556	1.50757
0.85	3.41155	13.0616	2.92464	1.59963
0.9	4.10951	16.1083	3.09067	1.73066
0.95	5.55085	22.6002	3.37897	1.96535
0.97	6.87482	28.7576	3.60033	2.15147

DRI values calculated at experimental points

Fa	Dose 6	Dose 6	DRI 6	DRI 6
0.439	1.53814	5.32517	2.56357	1.26790
0.526	1.77025	6.23856	2.21282	1.11403
0.717	2.46769	9.06909	2.46769	1.29558
0.8599	3.52295	13.5430	2.93579	1.61226
0.9497	5.53680	22.5358	3.46050	2.01213

0.9773	7.71427	32.7422	3.85714	2.33873

DRI Plot for Combo: 6 (6+6 [1:7])

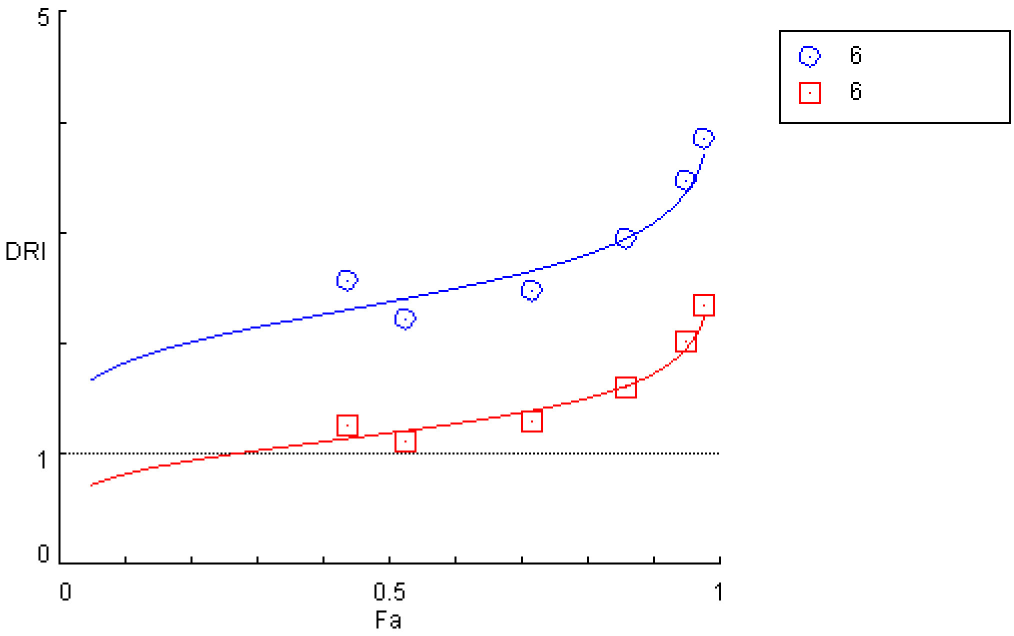

Log(DRI) Plot for Combo: 6 (6+6 [1:7])

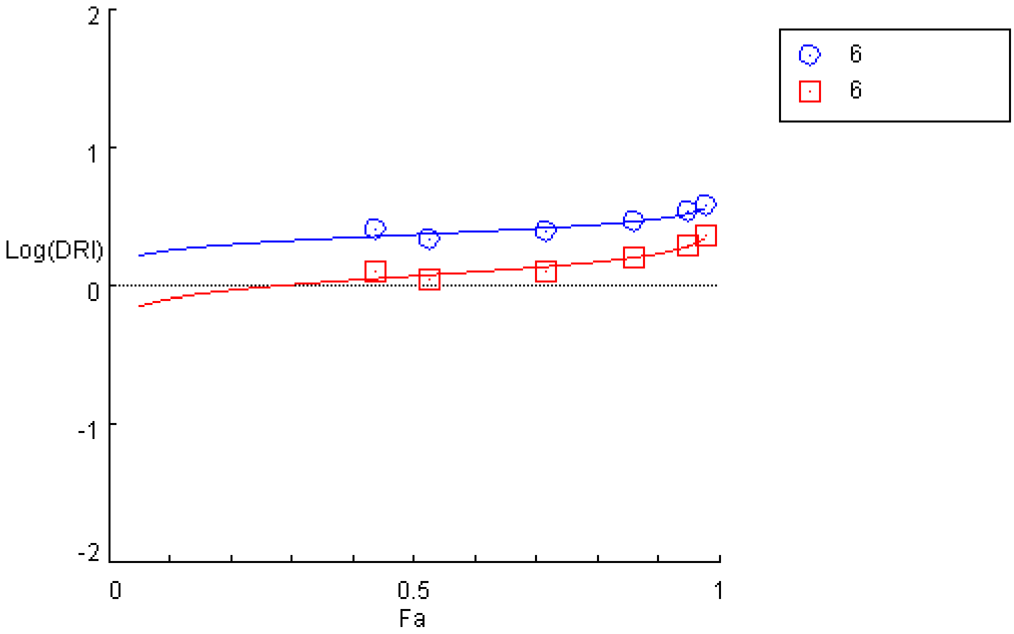

Isobologram for Combo: 6 (6+6 [1:7])

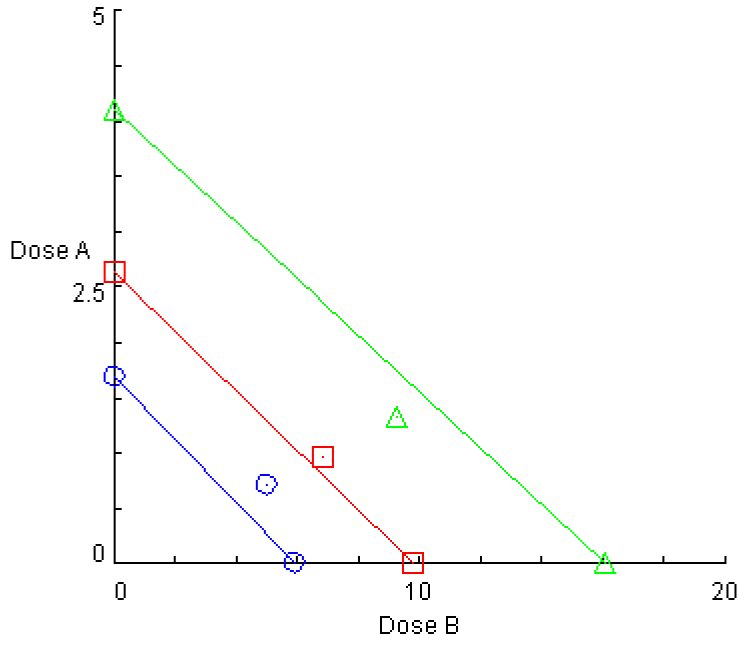

Polygonogram at Fa = 0.9

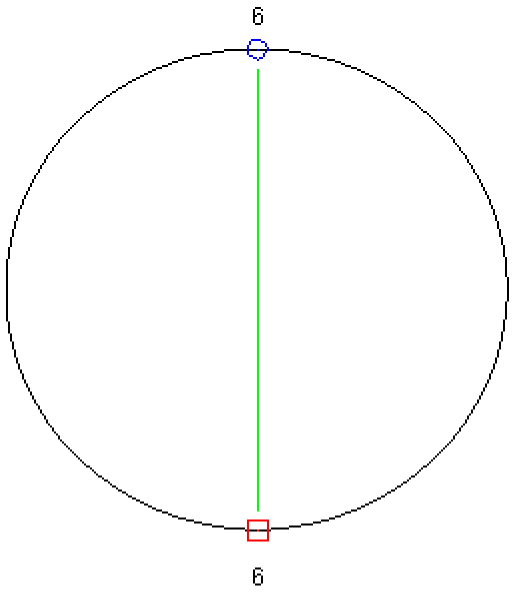

Summary Table

Experiment Name: SK5-LIANHE
Date: 2017/9/13
File Name: C:\Users\»¶»¶\Desktop\SK-5.cse
Description COMBINATION

Drug: vemurafenib (6) [uM/L]
Drug: GSK126 (6) [uM/L]
Drug Combo: combination (6) (6+6 [1:7])

Drug/Combo	Dm	m	r
6	1.69764	2.48537	0.97514
6	5.95110	2.20659	0.97749
6	5.71183	3.53355	0.98736

CI values at:

Combo	ED50	ED75	ED90	ED95
6	1.26039	1.06549	0.90137	0.80476

Data for Fa = 0.5

Drug/Combo	CI value	Dose 6	Dose 6
6		1.69764	
6			5.95110
6	1.26039	0.71398	4.99785

Data for Fa = 0.75

Drug/Combo	CI value	Dose 6	Dose 6
6		2.64130	
6			9.79090
6	1.06549	0.97434	6.82039

Data for Fa = 0.9

Drug/Combo	CI value	Dose 6	Dose 6
6		4.10951	
6			16.1083
6	0.90137	1.32965	9.30755

Data for Fa = 0.95

Drug/Combo	CI value	Dose 6	Dose 6
6		5.55085	
6			22.6002
6	0.80476	1.64276	11.4993

Data for Fa = 0.97

Drug/Combo	CI value	Dose 6	Dose 6
6		6.87482	
6			28.7576
6	0.74255	1.90950	13.3665
