# Supplementary material for: Identification of coexistence of BRAF V600E mutation and EZH2 gain specifically in melanoma as a promising target for combination therapy
Source: J Transl Med. 2017 Dec 4;15:243. doi: 10.1186/s12967-017-1344-z (PMC5716227; doi:10.1186/s12967-017-1344-z)
Supplement: Supplementary file 9 — Additional file 9: Figure S1. Sub-G0 cells detected in 2058 cells after combination therapy. Figure S2. Variations in apoptosis rate in all four BRAF V600E mutated cell lines after combination therapy. Figure S3. The levels of P-AKT at baseline and after treatment with GSK drug in all cell lines. [file 12967_2017_1344_MOESM9_ESM.docx]

**
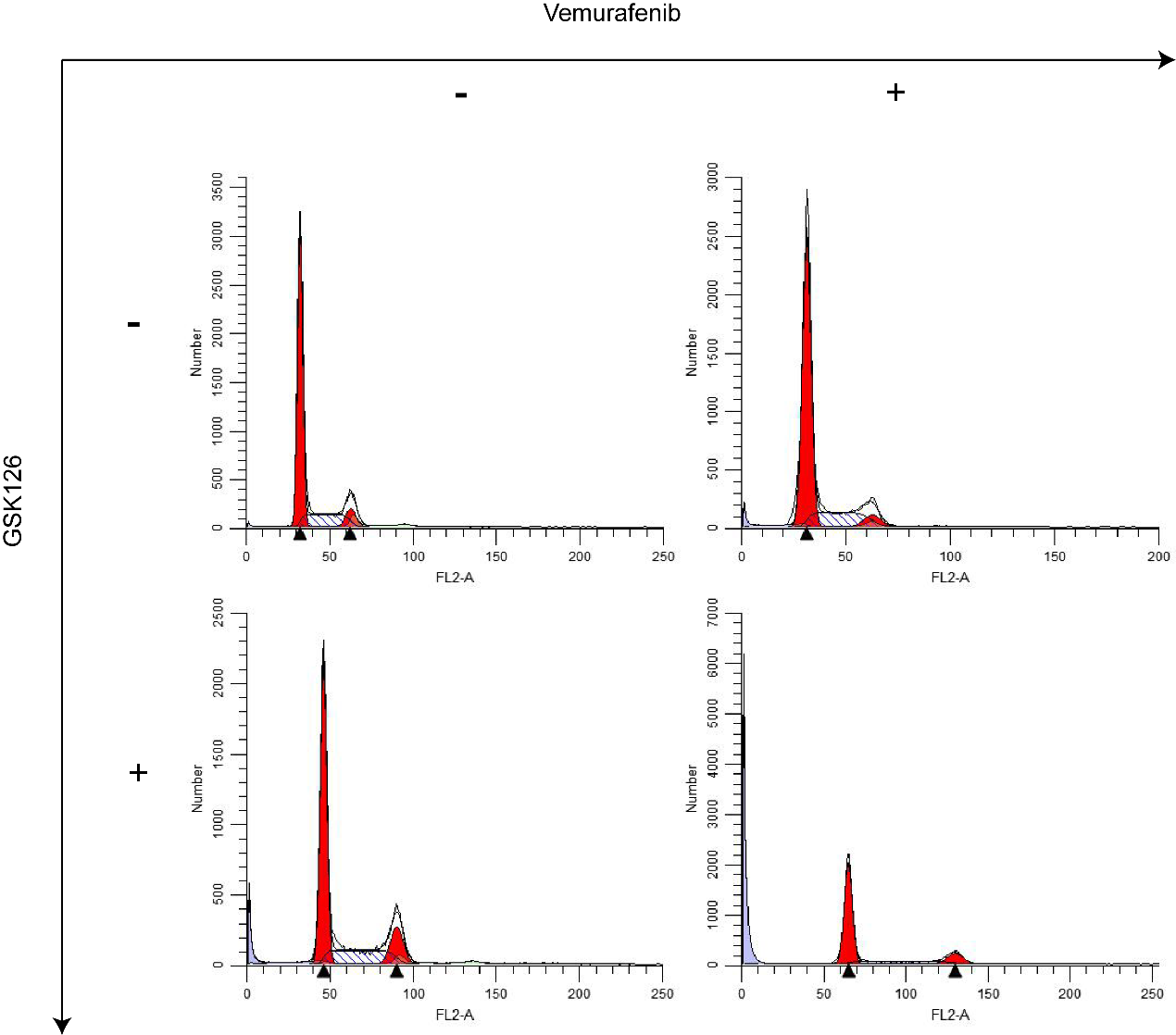
**

**Additional Figure S1. Sub-G0 cells detected in 2058 cells after combination therapy**

A2058 cells were treated with indicated concentrations of combination therapy and monotherapy for 72 h and subsequent to PI staining and flow cytometry analysis.


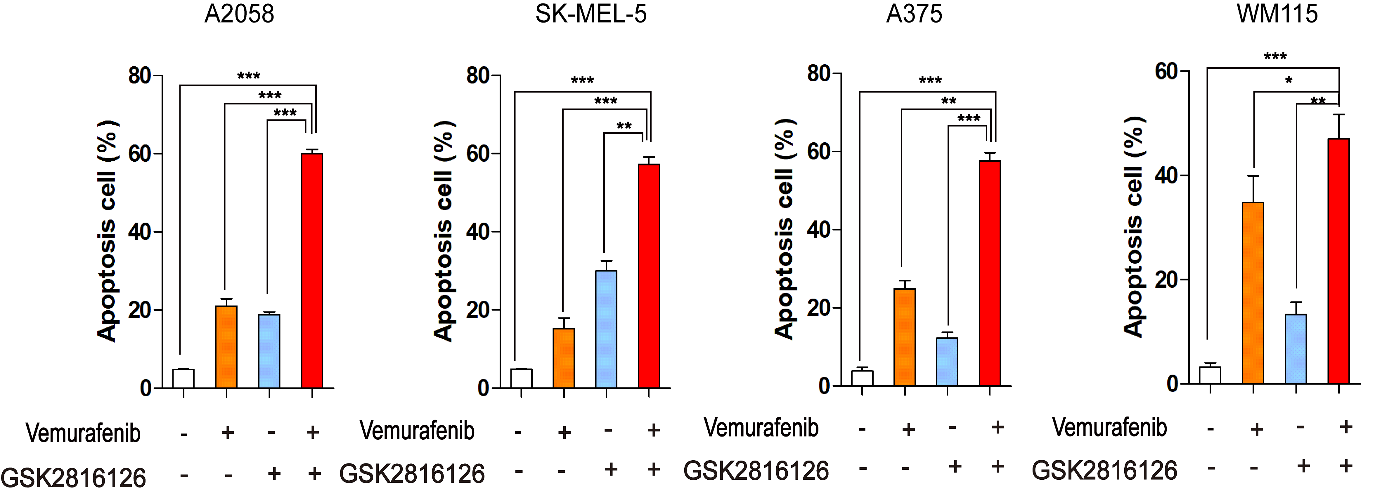


**Additional Figure S2. Variations in apoptosis rate in all four BRAF V600E mutated cell lines after combination therapy**

Cells were treated with indicating concentrations of combination therapy and monotherapy for 24 h. Apoptotic cells were detected with Annexin V-FITC and PI double staining followed by flow cytometry analysis. Graphs of the analysis were shown. Mean ± SD (n = 3).

Doses: A2058, vemurafenib and GSK126: 1uM each

SK-MEL-5, vemurafenib: 1uM, GSK126: 6uM

A375, vemurafenib: 1uM, GSK126: 10uM

WM 115, vemurafenib: 1uM, GSK126: 8uM


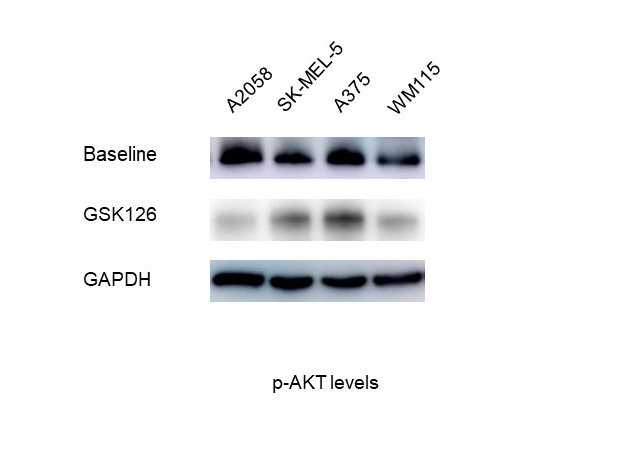


**Additional Figure S3. The levels of P-AKT at baseline and after treatment with GSK drug in all cell lines**

Immunoblots was conducted to determine the protein levels of P-AKT. GAPDH serves as loading control.
